# Bibliometric and visualized analysis of global distribution and research frontiers in tumor immune escape

**DOI:** 10.3389/fimmu.2025.1586120

**Published:** 2025-06-05

**Authors:** Chaihong Zhang, Lihong Chen

**Affiliations:** Department of Obstetrics and Gynecology, Shaanxi Provincial People’s Hospital, Xi’an, Shaanxi, China

**Keywords:** immune escape, tumor, immunotherapy, VOSviewer, biblioshiny, CiteSpace

## Abstract

**Background:**

Tumor cells employ various mechanisms to evade detection and attack by the immune system, a phenomenon known as tumor immune escape, which represents a significant target for immunotherapy. Both primary and secondary immune escape mechanisms pose substantial challenges that hinder the efficacy of immunotherapy. This study aims to systematically examine the knowledge structure, hotspot frontiers, emerging trends, and future directions in the field of tumor immune escape through the application of bibliometric methods and knowledge mapping analysis.

**Methods:**

A comprehensive search of the Web of Science Core Collection (WoSCC) was conducted for publications pertaining to tumor immune escape from January 1, 2015, to November 30, 2024. The annual publication data retrieved from the WoSCC were analyzed utilizing Microsoft Office Excel 2019. Furthermore, bibliometric analysis and visualization were executed using VOSviewer, Biblioshiny, and CiteSpace.

**Results:**

This study encompassed a total of 11,128 articles published across 1,612 journals, authored by 71,684 individuals affiliated with 9,254 institutions in 121 countries. The United States, China, and Germany emerged as the leading contributors to this field, collectively accounting for 79.99% of all publications. Notable international collaboration was observed between the United States and China. Frontiers in Immunology, Nature Communications, the Journal for ImmunoTherapy of Cancer, and Nature were identified as the four most influential journals in tumor immune escape research. Zhang Wei was noted for the highest publication output, while Freeman Gordon J achieved the highest citation rate. Fudan University was recognized as the most productive institution, whereas Harvard Medical School was acknowledged as the most cited institution. Current hotspot frontiers in tumor immune escape research include immunotherapy, the tumor microenvironment, PD-L1, and PD-1. Additionally, emerging frontiers in recent years encompass immune checkpoint inhibitors, immune infiltration, natural killer cells, extracellular vesicles, immunogenic cell death, metabolism, ferroptosis, melanoma, lung adenocarcinoma, and prognosis.

**Conclusion:**

A comprehensive investigation into the mechanisms of tumor immune escape is essential for overcoming the existing challenges in immunotherapy. This study systematically analyzes the current state, research frontiers, and future directions, identifying the most prolific and highly cited documents, journals, authors, institutions, and countries in the field of tumor immune escape.

## 1 Introduction

Immunotherapy, a revolutionary breakthrough in cancer treatment, has transitioned from directly eradicating tumors to activating the immune system. This paradigm shift has fundamentally altered the landscape of cancer therapy, heralding a new era in oncological treatment (1). The primary objectives of immunotherapy include achieving durable remission, long-term survival (2), and potentially curing cancer (3). Its extensive anti-tumor activity (4), effectiveness against drug-resistant tumors (5), and capacity to facilitate personalized treatment (6) have solidified its role as an essential element of cancer therapy. However, cancer immunotherapy still faces challenges related to tumors, treatments, patients, and technology. Tumor-related challenges include tumor heterogeneity (7) and immune escape (8). Treatment-related challenges encompass adverse reactions (9), high costs (10), and treatment resistance resulting from tumor immune escape (11). Patient-related challenges pertain to differences in patients’ immune systems (12) and genetic backgrounds (13). Technology-related challenges involve difficulties in target selection (14) and limitations in drug delivery systems (15).

Tumor immune escape is a particularly critical challenge in cancer immunotherapy, as it directly undermines the core mechanism of activating the immune system to recognize and eliminate cancer cells (16). This phenomenon refers to the various strategies utilized by tumor cells to evade recognition and attack by the immune system, thereby allowing their survival and proliferation *in vivo* (17). The primary mechanisms of tumor immune escape include tumor antigen deletion (16), antigen presentation and recognition abnormalities (18), immune cell exhaustion and dysfunction (16), activation of inhibitory immune checkpoints (18, 19), increased immunosuppressive cells and proteins (16, 19), alterations in the immunosuppressive microenvironment (18, 19), and metabolic reprogramming (16, 18). A comprehensive investigation of tumor immune escape mechanisms is of significant scientific and clinical importance, as it can inform the development of more effective immunotherapy strategies, identify novel targets for immunotherapy, enhance the efficacy of existing treatments, overcome resistance to immunotherapy, optimize combination therapy approaches, improve patient outcomes, and reduce treatment costs.

Bibliometrics is the most developed and widely applied branch of scientometrics (20), with medical bibliometrics serving as a specialized subset focused on the medical field (21). Medical bibliometrics utilizes mathematical and statistical methodologies to analyze the characteristics, distribution, and evolutionary patterns of medical literature (22). Tools for bibliometric analysis, such as VOSviewer (23), Biblioshiny (24), and CiteSpace (25), facilitate the scientific analysis of medical literature and the visualization of results. VOSviewer is bibliometric software designed for constructing and visualizing scientometric networks and knowledge maps, primarily used for co-authorship, co-occurrence, citation, bibliographic coupling, and co-citation analyses (23). The Biblioshiny online platform is an interactive application built on the R programming language (https://www.R-project.org) and the Bibliometrix package (https://www.bibliometrix.org), specifically designed for comprehensive science mapping analysis and result visualization (24). CiteSpace is a significant tool for bibliometric analysis and literature visualization based on Java (https://www.java.com), primarily used for centrality, clustering, timeline view, and citation burst analysis (25). Through these analyses, it is possible to quantitatively evaluate the research contributions and academic influence of medical researchers, institutions, countries, and journals. Additionally, it helps identify research hotspots, emerging frontiers, and innovation trends within the medical field, as well as in planning future directions for medical research (22). Furthermore, medical bibliometrics provides a scientific basis for the formulation of medical research policies, public health initiatives, and clinical practice guidelines. It plays a crucial role in optimizing funding and resource allocation, fostering academic exchanges and international collaboration, conducting disease surveillance, promoting technological innovation, enhancing clinical translation, and improving healthcare quality. For example, during the COVID-19 pandemic, medical bibliometrics facilitated the rapid identification of research hotspots and collaboration networks, providing essential support for epidemic prevention and control (26). Nevertheless, there is currently a lack of bibliometric analyses regarding the literature on tumor immune escape. Although the volume of relevant research articles has consistently increased, the knowledge maps, research hotspots, emerging frontiers, and innovation trends in this field remain poorly defined. This study employs VOSviewer, Biblioshiny, and CiteSpace to conduct a bibliometric analysis of the literature on tumor immune escape from 2015 to 2024. The objective is to identify key contributors, research hotspots, and emerging frontiers, while also exploring future innovation trends and development prospects.

## 2 Materials and methods

### 2.1 Data collection and search strategies

**Figure 1:**
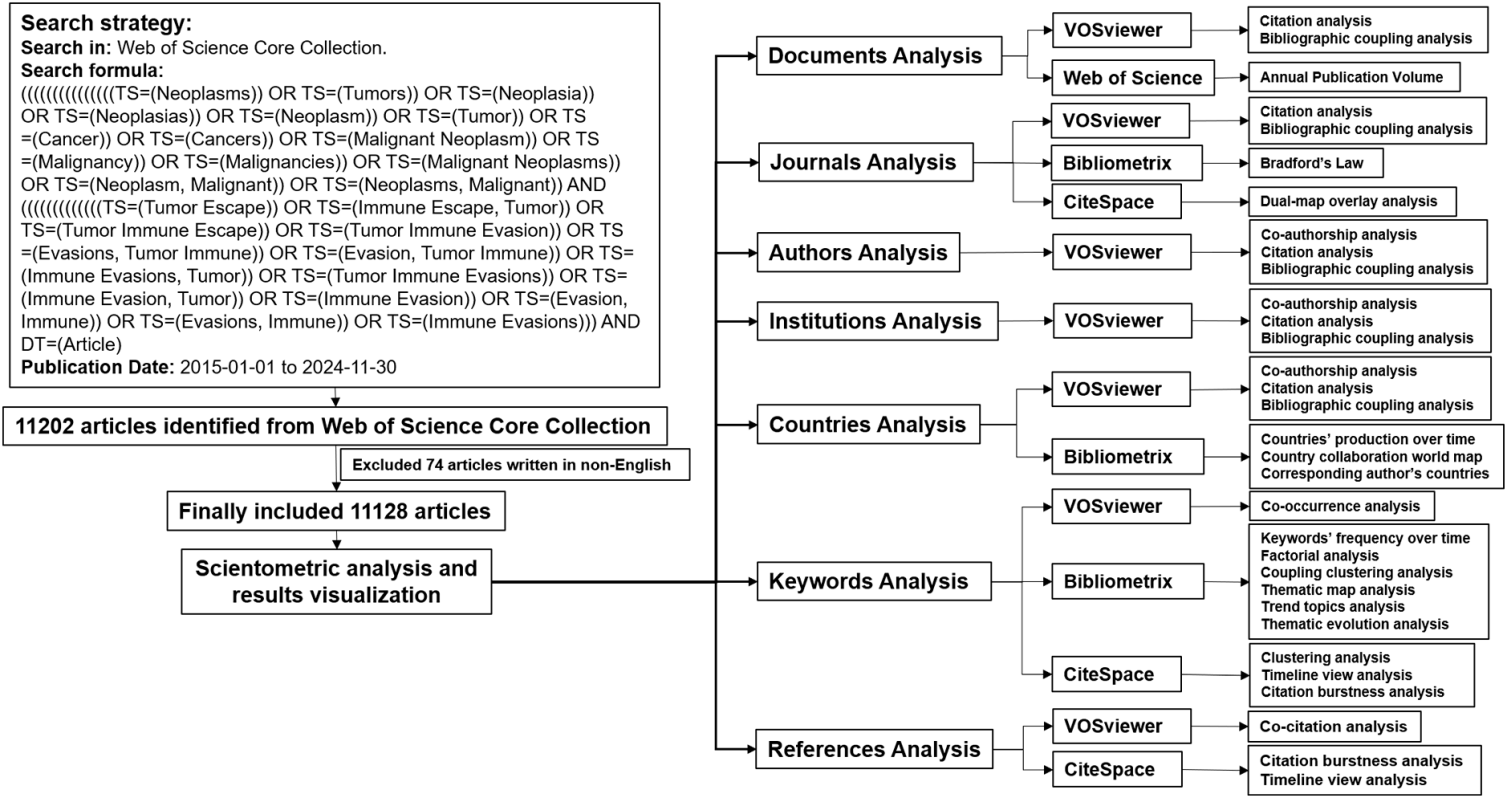
The flowchart illustrates the process of literature screening and bibliometric analysis.

A systematic and comprehensive search of the Web of Science Core Collection (WoSCC) database (https://webofscience.clarivate.cn/wos/woscc/advanced-search) was conducted on December 14, 2024, to identify publications pertaining to tumor immune escape from January 1, 2015, to November 30, 2024. The inclusion criteria for this search were as follows: (1) studies focused on tumor immune escape; (2) articles published in the English language; (3) document type limited to original research articles; (4) publications sourced from the WoSCC; and (5) the search period extended from January 1, 2015, to November 30, 2024. Conversely, the exclusion criteria included: (1) studies unrelated to tumor immune escape; (2) articles not written in English; (3) documents not categorized as original research articles; and (4) duplicate records. The search formula is detailed in **Figure 1**. After excluding non-English publications, a total of 11,128 articles were identified. The “Full Record and Cited References” for each article were subsequently exported as plain text files. The data collection and screening process were carried out by two researchers. A flowchart illustrating the literature screening and bibliometric analysis is presented in **Figure 1**. The search formula is in **Supplementary File 1**.

### 2.2 Bibliometric analysis and visualization

To analyze the annual publication data from the WoSCC, Microsoft Office Excel 2019 (Microsoft, Redmond, WA, USA) was utilized. According to the annual publication data, we calculated the cumulative annual publications and then performed a linear fitting analysis. Based on this analysis, we drew the trend line and displayed the calculated formula along with the R-squared value. Additionally, VOSviewer (23), Biblioshiny (24), and CiteSpace (25) were employed for bibliometric analysis and visualization.

#### 2.2.1 VOSviewer analysis

VOSviewer (version 1.6.20) is a bibliometric tool designed for constructing and visualizing scientometric networks and knowledge maps (23). In this study, VOSviewer was primarily employed to conduct analyses on documents, journals, cited journals, authors, cited authors, institutions, countries, author keywords, and references. Researchers generated co-authorship, keyword co-occurrence, citation, bibliographic coupling, or co-citation maps based on bibliographic data. The parameters were set as follows: choose type of data: create a map based on bibliographic data; choose data source: read data from bibliographic database files; supported file types: Web of Science; visualization: network visualization; weights: documents/citations; font: Open Sans; colors: different clusters were designated with distinct colors. In the maps, node sizes reflect the number or frequency of relevant elements, line widths between nodes indicate interaction strength, and colors signify different clusters. Detailed parameter settings can be found in **Supplementary Table 1**.

VOSviewer was utilized to visualize co-authorship networks among authors, institutions, or countries. The parameters were set as follows: counting method: full counting; minimum number of documents of an author, institution, or country: 1; minimum number of citations of an author, institution, or country: 0. This analysis identified 71,684 authors, 9,254 institutions, and 121 countries. For each identified entity, the total strength of co-authorship links with other entities was calculated, leading to the selection of the top ten authors, institutions, or countries based on the highest publication and citation counts.

Additionally, VOSviewer was employed to visualize a co-occurrence network of author keywords. The parameters were set as follows: counting method: full counting; minimum number of occurrences of an author keyword: 1. This analysis identified 16,465 author keywords, and for each keyword, the total strength of co-occurrence links with other keywords was computed. The thresholds for the selection of the top 25 author keywords were specified as follows: the minimum number of occurrences for a keyword was set at 100, and the minimum total link strength for a keyword was set at 500.

Furthermore, VOSviewer facilitated the visualization of citation networks for documents, journals, authors, institutions, or countries. The parameter for document analysis was set as follows: minimum number of citations of a document: 0. This analysis identified a total of 11,128 documents. For each document, the number of citation links was calculated, allowing for the selection of the top ten documents with the highest global or local citations. For journal, author, institution, or country analyses, the parameters were set as follows: minimum number of documents for a journal, author, institution, or country: 1; minimum number of citations for a journal, author, institution, or country: 0. This analysis identified 1,612 journals, 71,684 authors, 9,254 institutions, and 121 countries. The total strength of citation links for each entity was computed, leading to the selection of the top fifteen journals and the top ten authors, institutions, or countries with the highest publication and citation counts.

Moreover, VOSviewer was utilized to visualize bibliographic coupling networks for documents, journals, authors, institutions, or countries. The parameters of document analysis were set as follows: counting method: full counting; minimum number of citations of a document: 0. This analysis identified a total of 11,128 documents. The total strength of bibliographic coupling links for each document was calculated, allowing for the selection of the top ten documents with the highest global or local citations. For journal, author, institution, or country analyses, the parameters were set as follows: counting method: full counting; minimum number of documents of a journal, author, institution, or country: 1; minimum number of citations of a journal, author, institution, or country: 0. This analysis identified 1,612 journals, 71,684 authors, 9,254 institutions, and 121 countries. The total strength of bibliographic coupling links for each entity was computed, resulting in the selection of the top fifteen journals and the top ten authors, institutions, or countries with the highest publication and citation counts.

Lastly, VOSviewer was employed to visualize co-citation networks for cited references, cited journals, or cited authors. The parameters were set as follows: counting method: full counting; minimum number of citations of a cited reference, cited source, or cited author: 1. This analysis identified 331,872 cited references, 14,355 cited journals, and 174,657 cited authors. For each cited entity, the total strength of co-citation links with other entities was calculated, resulting in the selection of the top fifteen cited journals, top ten cited references, and top ten cited authors with the highest citation counts.

#### 2.2.2 Biblioshiny analysis

Biblioshiny (https://www.bibliometrix.org/home/index.php/layout/biblioshiny) is a Shiny application that provides a web interface platform for bibliometrix, which is based on R software (version 4.4.2) and the bibliometrix package (version 4.3.2) (24). In this study, Biblioshiny was primarily employed to conduct analyses on journals, countries, and author keywords. The parameters were set as follows: choose what to do: import raw file; database: Web of Science; author name format: full name; choose a file: download.txt.

Biblioshiny was applied to conduct an analysis based on Bradford’s Law concerning the distribution of journals that focus on tumor immune escape, with the shaded area representing the core journals. Additionally, Biblioshiny was utilized to examine the distribution of corresponding authors’ countries, with a limitation of 10 countries. In addition, Biblioshiny was employed to analyze the output of different countries over time, with a restriction to 3 countries. The application also facilitated the visualization of a world map depicting collaboration among countries, with parameters set as follows: method parameter: minimum edges = 50; graphical parameter: edge size = 5.

Moreover, Biblioshiny was utilized to assess the frequency of keywords over time, with parameters set as follows: field: author’s keywords; occurrences: cumulative; number of keywords: 7. The application was also employed to conduct a coupling clustering analysis, utilizing the following parameters: unit of analysis: documents; coupling measured by: references; impact measure: local citation score; cluster labeling by: author’s keywords; number of units: 250; labels per cluster: 3; clustering algorithm: Walktrap. Additionally, a trend topics analysis was executed using Biblioshiny, with parameters set as follows: field: author’s keywords; timespan: 2015-2024; minimum keyword frequency: 50; number of keywords per year: 3. Louvain is a clustering algorithm designed for the analysis of large-scale nodes and is unable to form semantic labels. We chose Walktrap as the clustering algorithm because it has high accuracy and is suitable for analyzing small-scale nodes.

Furthermore, Biblioshiny facilitated a thematic evolution analysis, with the following parameters: field: author’s keywords; number of keywords: 250; minimum cluster frequency (per thousand documents): 5; weight index: inclusion index weighted by keyword occurrences; clustering algorithm: Walktrap; time slices: number of cutting points: 1; cutting year 1: 2021. Lastly, a thematic map analysis was conducted using Biblioshiny, with parameters set as follows: field: author’s keywords; number of keywords: 250; minimum cluster frequency (per thousand documents): 2; clustering algorithm: Walktrap. A factorial analysis was also performed using Biblioshiny, with the following parameters: method: multiple correspondence analysis; field: author’s keywords; number of terms: 25; number of clusters: 3.

#### 2.2.3 CiteSpace analysis

CiteSpace (version 6.1.6) is a significant tool for bibliometric analysis and literature visualization. In this study, CiteSpace was primarily utilized to perform analyses on cited journals, cited authors, journals, keywords, and references. The parameters were set as follows: link strength: cosine; link scope: within slices; time slicing: from January 2015 to November 2024; years per slice: 1.

CiteSpace was employed to compute the centrality of cited journals and authors, with centrality being a vital metric for identifying and assessing the importance of these entities. The parameters were set as follows: node types: cited journal/cited author; selection criteria: select the top 10 levels of the most cited or occurring items from each slice; each level may include multiple qualified nodes.

Additionally, CiteSpace was utilized to visualize the dual-map overlay of journals, with the following parameters: overlay maps: journal citation reports (JCR) maps; label top N journals: 10; the method of standardization: z-score; α: 1; source circle size: 10; target circle size: 5; snap to centroids: 0.

Furthermore, CiteSpace facilitated keyword analyses, with parameters including: node types: keyword; selection criteria: select the top 100 levels of the most cited or occurred items from each slice; each level may include multiple qualified nodes. The parameters for cluster and timeline analyses were as follows: clusters: find clusters; label clusters with: indexing terms; show cluster labels by: log-likelihood ratio (LLR); toggle: nodes colored by cluster membership; visualization: timeline view. For burstness analysis, the parameters included: the number of states: 2; minimum duration: 2; burst items found: 0; how many keywords have citation bursts: 78; how many keywords to be included: 25. Louvain is a topology-driven algorithm and is unable to handle text semantic information to form semantic labels for naming clusters. We chose the Log-Likelihood Ratio (LLR) as the clustering naming method because it can identify the significantly high-frequency terms in the clusters and form semantic labels.

Lastly, CiteSpace was employed to analyze references, with parameters set as follows: node types: reference; selection criteria: select the top 100 levels of the most cited or occurred items from each slice; each level may include multiple qualified nodes. The parameters for timeline analysis included: clusters: find clusters; label clusters with: indexing terms; show cluster labels by: log-likelihood ratio; visualization: timeline view. For burstness analysis, the parameters included: the number of states: 2; minimum duration: 2; burst items found: 0; how many references have citation bursts: 242; how many references are to be included: 25.

### 2.3 Ethics in research

This study did not require ethical approval as it involved no human or animal experiments. The data were obtained from publicly accessible databases, and their use did not raise any ethical concerns.

## 3 Results

### 3.1 Analysis of documents

#### 3.1.1 Annual growth trend of articles in the field of tumor immune escape

**Figure 2:**
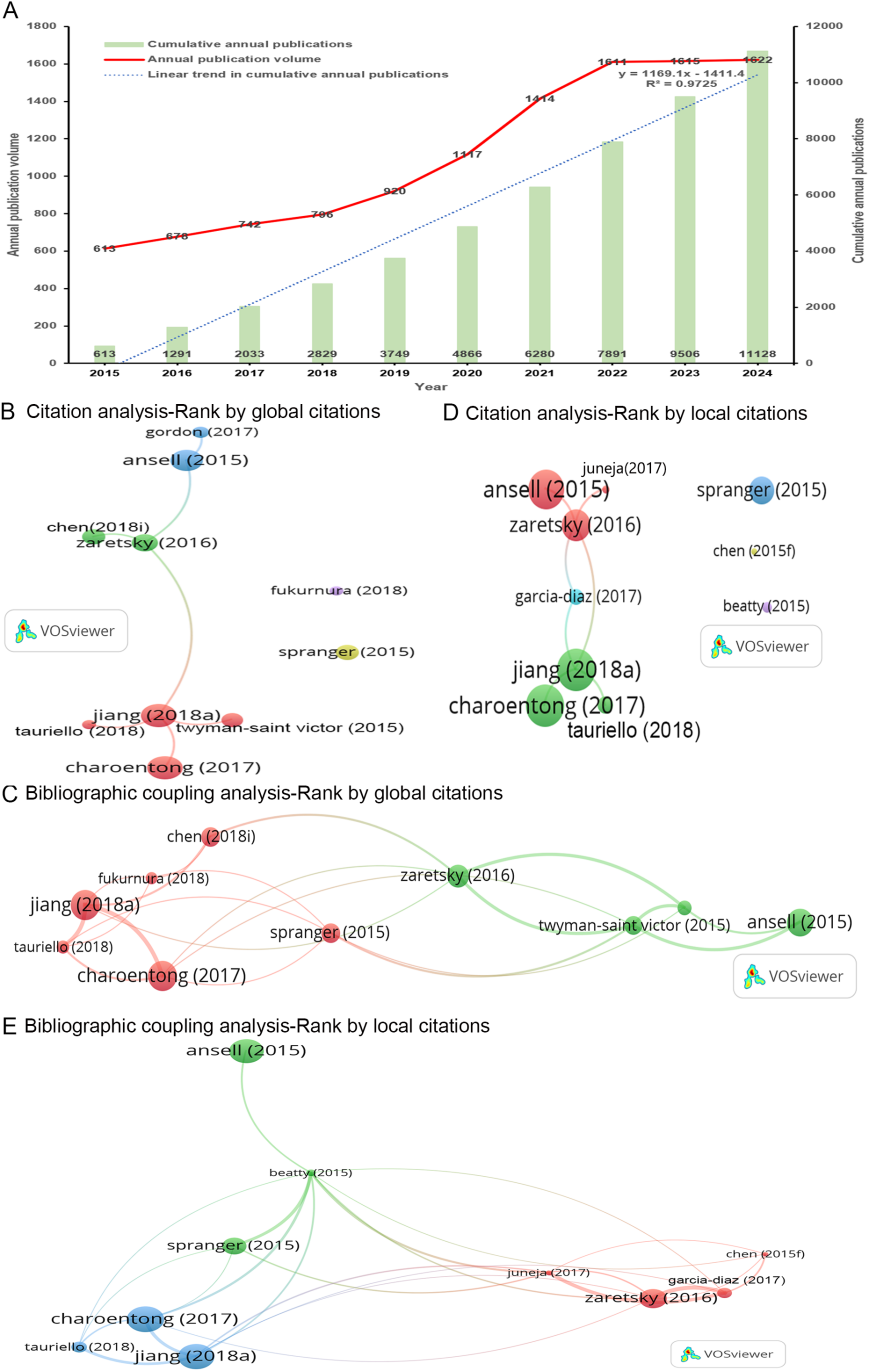
Analysis of documents. **(A)** The annual and cumulative growth trends of publications concerning tumor immune escape over time are presented. The annual publication volume is represented by the red line, indicating the number of publications per year. The cumulative annual publications are depicted by the green column, which reflects the total number of publications up to each respective year. The blue dotted line illustrates the trend-fitted curves derived from a linear regression model. Data sourced from Web of Science. **(B-E)** The size of the nodes corresponds to citation frequency, while the width of the lines indicates the strength of interactions, with colors representing distinct clusters. **(B, D)**. Two network visualization maps depict the citation analysis of the ten most cited articles on a global and local scale, respectively. **(C, E)** Two network visualization maps illustrate the bibliographic coupling analysis of the ten most cited articles on a global and local scale, respectively.

A total of 11,128 articles related to tumor immune escape were published from January 1, 2015, to November 30, 2024. As illustrated in **Figure 2A**, there has been a consistent annual increase in the number of publications related to this topic. Notably, between 2019 and 2022, there was a significant surge in publication output, with numbers escalating from 920 to 1,611. Furthermore, from 2022 to 2024, the output consistently surpassed 1,600, reaching a peak of 1,622 in 2024. The blue dotted line in the figure represents a linear fitting curve (R² = 0.97), which effectively captures the annual growth trend of cumulative publications in this field.

#### 3.1.2 Most globally cited documents

Global citation counts concentrate on the total number of citations recorded in the Web of Science database. **Table 1** shows the ten articles with the highest global citation counts. Notably, five of these articles have surpassed 2,000 citations, with Charoentong (2017) being the most globally cited, followed by Jiang (2018a), Ansell (2015), Zaretsky (2016), and Spranger (2015) (**Supplementary Table 2**).

**Table 1:** Top ten most cited articles in tumor immune escape research.

Top ten most globally cited articles								Top ten most locally cited articles							
First Author (Date)	GC	LC	LC/GC	NGC	NLC	TLCA	TLSBCA	First Author (Date)	GC	LC	LC/GC	NGC	NLC	TLCA	TLSBCA
**charoentong** (2017)	3040	169	5.56	47.02	44.06	**170**	**2551**	**jiang (2018a)**	3009	251	8.34	43.71	71.36	**257**	**3565**
**jiang (2018a)**	3009	251	8.34	43.71	71.36	**257**	**3565**	**zaretsky** (2016)	2282	203	8.9	34.05	55.27	**204**	**2178**
ansell (2015)	2805	127	4.53	42	37.61	127	1247	**charoentong** (2017)	3040	169	5.56	47.02	44.06	**170**	**2551**
**zaretsky** (2016)	2282	203	8.9	34.05	55.27	**204**	**2178**	garcia-diaz (2017)	1197	155	12.95	18.51	40.41	156	2099
spranger (2015)	2004	150	7.49	30.01	44.42	150	1343	spranger (2015)	2004	150	7.49	30.01	44.42	150	1343
chen (2018i)	1988	101	5.08	28.88	28.71	104	1777	ansell (2015)	2805	127	4.53	42	37.61	127	1247
twyman-saint victor (2015)	1854	46	2.48	27.76	13.62	46	1989	beatty (2015)	783	124	15.84	11.72	36.72	124	2877
gordon (2017)	1511	83	5.49	23.37	21.64	85	2077	tauriello (2018)	1287	122	9.48	18.7	34.68	122	1220
tauriello (2018)	1287	122	9.48	18.7	34.68	122	1220	chen (2015f)	540	114	21.11	8.09	33.76	114	1515
fukurnura (2018)	1278	39	3.05	18.57	11.09	41	1496	juneja (2017)	600	106	17.67	9.28	27.64	109	2859

GC, Global Citations; LC, Local Citations; LC/GC, LC/GC Ratio (%); NGC, Normalized Global Citations; NLC, Normalized Local Citations; TLCA, Total links of citation analysis; TLSBCA, Total link strength of bibliographic coupling analysis.

Bold values represent the top three values in each item.

The citation analysis primarily examines the reciprocal citation relationships among 11,128 local articles. A citation network was constructed based on the ten most globally cited articles (**Figure 2B**). Charoentong (2017) emerged as the most globally cited article (**Table 1**). Within this network, Charoentong (2017) exhibited a mutual citation relationship solely with Jiang (2018a) (link = 1) (**Figure 2B**). The three articles with the highest number of links in the citation analysis were Jiang (2018a), Zaretsky (2016), and Charoentong (2017) (**Table 1**).

The bibliographic coupling analysis investigates the citation of shared references across the 11,128 articles. A bibliographic coupling network was established based on the ten most globally cited articles (**Figure 2C**). Among these articles, Charoentong (2017) shared the same references with Jiang (2018a) (links = 4), Tauriello (2018) (links = 2), Spranger (2015) (link = 1), Zaretsky (2016) (link = 1), and Twyman-Saint Victor (2015) (link = 1) (**Figure 2C**). The three articles with the highest total link strength in the bibliographic coupling analysis were Jiang (2018a), Charoentong (2017), and Zaretsky (2016) (**Table 1**).

#### 3.1.3 Most locally cited documents

Local citation counts emphasize the number of citations within the 11,128 local articles. **Table 1** outlines the top ten articles according to local citation counts. Among these articles, two have received over 200 citations, with Jiang (2018a) emerging as the most locally cited article, followed by Zaretsky (2016).

A citation network was constructed for the ten most locally cited articles (**Figure 2D**). Jiang (2018a) was recognized as the most locally cited article (**Table 1**). Within this network, Jiang (2018a) exhibited mutual citation relationships with Charoentong (2017) (link = 1), Tauriello (2018) (link = 1), Zaretsky (2016) (link = 1), and Garcia-Diaz (2017) (link = 1) (**Figure 2D**). The citation analysis identified Jiang (2018a), Zaretsky (2016), and Charoentong (2017) as the three articles with the most links (**Table 1**).

Additionally, a bibliographic coupling network was established based on the ten most locally cited articles (**Figure 2E**). Among these articles, Jiang (2018a) shared the same references with Charoentong (2017) (links = 4), Tauriello (2018) (links = 4), Zaretsky (2016) (link = 1), Juneja (2017) (links = 2), Garcia-Diaz (2017) (link = 1), and Beatty (2015) (links = 2) (**Figure 2E**). The bibliographic coupling analysis identified Jiang (2018a), Charoentong (2017), and Zaretsky (2016) as the three articles with the highest total link strength (**Table 1**).

In summary, Jiang (2018a), Charoentong (2017), and Zaretsky (2016) are the three most important articles in the field of tumor immune escape. Jiang (2018a) and Charoentong (2017) are situated within the same cluster and exhibit a stronger correlation (**Figures 2B–E**). Jiang (2018a), published in Nature Medicine (Impact Factor = 58.7, Q1), has co-authors primarily affiliated with the Dana-Farber Cancer Institute in the United States of America (USA). Charoentong (2017), published in Cell Reports (Impact Factor = 7.5, Q1), has co-authors mainly from the Medical University of Innsbruck in Austria. Zaretsky (2016), published in the New England Journal of Medicine (Impact Factor = 96.2, Q1), has co-authors mainly from the University of California, Los Angeles, in the USA.

### 3.2 Analysis of journals

#### 3.2.1 Bradford’s Law analysis

**Figure 3:**
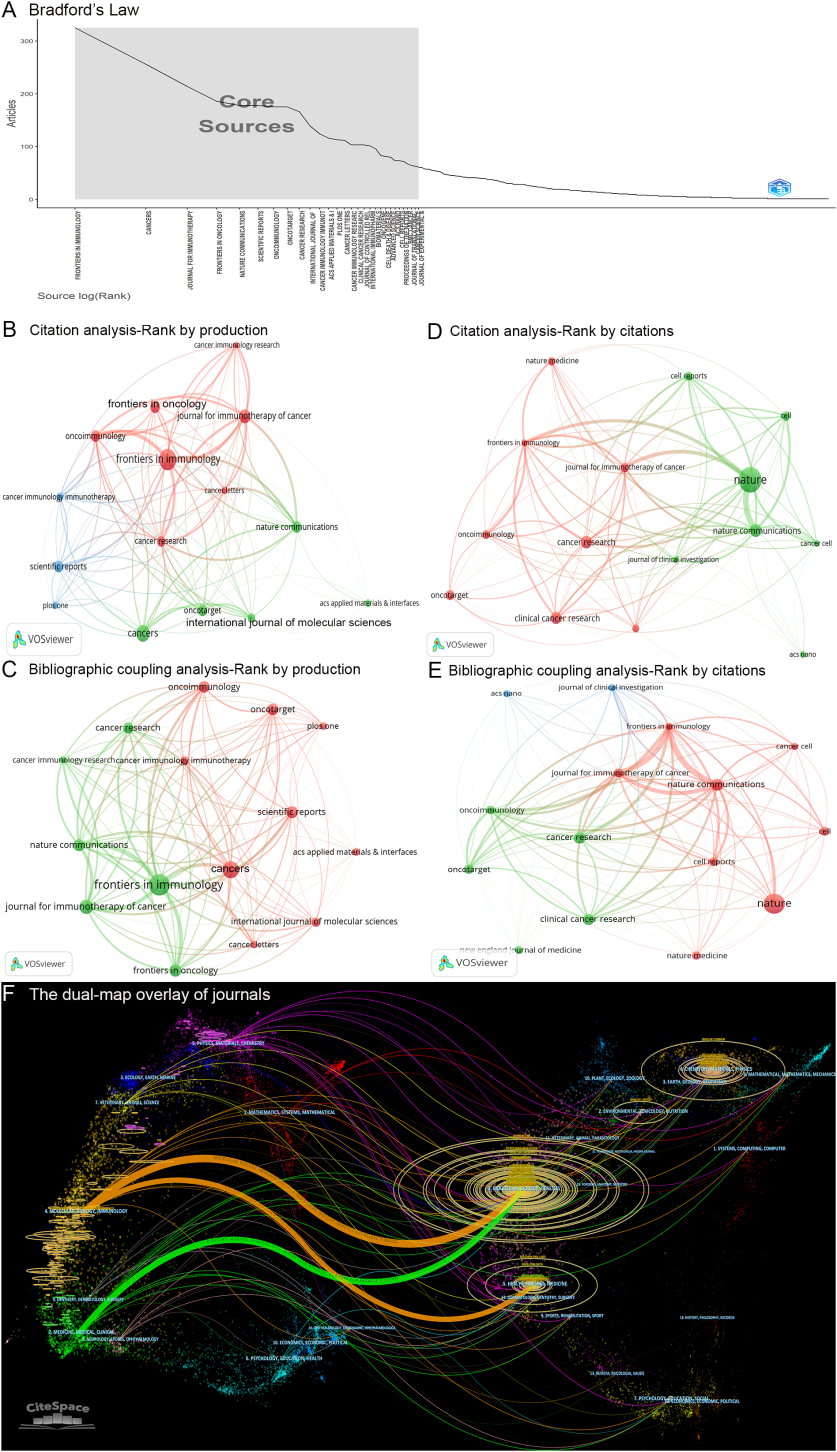
Analysis of journals. **(A)** Distribution of core journals in accordance with Bradford’s Law. **(B, C)** Two network maps that illustrate the citation and bibliographic coupling analyses of the fifteen most productive journals, respectively. Node size reflects the number of articles, line width indicates interaction strength, and colors signify different clusters. **(D, E)** Two network maps that depict the citation and bibliographic coupling analyses of the fifteen most cited journals, respectively. Node size indicates citation frequency, line width shows interaction strength, and colors denote clusters. **(F)** The dual-map overlay of journals illustrates the distribution of journal disciplines, with the left side representing the citing field and the right side representing the cited field. The citation curve indicates the flow of knowledge from the citing domain to the cited domain, with colors representing various disciplines. Parameters: the method of standardization: z-score; α: 1; source circle size: 10; target circle size: 5; snap to centroids (<Radius): 0.

Using Bradford’s Law, the core journals in tumor immune escape were identified, such as Frontiers in Immunology, Cancers, the Journal for ImmunoTherapy of Cancer, Frontiers in Oncology, Nature Communications, and Scientific Reports (**Figure 3A**). According to Bradford’s Law, a total of 29 core journals have been identified. The subsequent analysis will focus on 50% of these core journals, specifically the top 15 ranked publications.

#### 3.2.2 Most productive journals

A total of 11,128 articles related to tumor immune escape were published across 1,612 different journals. Among these journals, Frontiers in Immunology was identified as the leading journal with the highest publication volume (n = 325, 2.92%), followed by Cancers (n = 256, 2.30%) and the Journal for ImmunoTherapy of Cancer (n = 214, 1.92%). Collectively, the top 15 most productive journals contributed 2,560 articles, accounting for 23.01% of the total 11,128 articles (**Table 2**).

**Table 2:** Top 15 journals in tumor immune escape research.

Top 15 most productive journals						Top 15 most cited journals						Top 15 most co-cited journals				
Journals	Counts	IF 2023	Q 2023	TLSCA	TLSBCA	Journals	Citations	IF 2023	Q 2023	TLSCA	TLSBCA	Journals	Citations	IF 2023	Q 2023	Centrality
**frontiers in immunology**	325	5.7	Q1	**938**	**307807**	**nature**	19688	50.5	Q1	**1360**	58815	**nature**	15906	50.5	Q1	**0.16**
cancers	256	4.5	Q1	544	185485	**nature communications**	10290	14.7	Q1	**1065**	**220944**	**cancer research**	15288	12.5	Q1	**0.50**
**journal for immunotherapy of cancer**	214	10.3	Q1	**790**	**194094**	cancer research	9651	12.5	Q1	778	133497	pnas	12210	9.4	Q1	0.15
frontiers in oncology	186	3.5	Q2	409	165365	clinical cancer research	9494	10.4	Q1	691	103545	cell	11873	45.6	Q1	0.13
**nature communications**	178	14.7	Q1	**1065**	**220944**	oncotarget	7674	—	—	473	116304	**clinical cancer research**	10836	10.4	Q1	**0.16**
scientific reports	178	3.8	Q1	380	118599	**journal for immunotherapy of cancer**	7399	10.3	Q1	790	**194094**	journal of immunology	9396	3.6	Q2	0.04
oncoimmunology	175	6.5	Q1	676	150059	cell	7334	45.6	Q1	513	50507	science	9080	44.8	Q1	0.01
oncotarget	175	—	—	473	116304	cell reports	7127	7.5	Q1	677	92828	blood	9046	21.1	Q1	0
cancer research	166	12.5	Q1	778	133497	oncoimmunology	6855	6.5	Q1	676	150059	nature communications	8951	14.7	Q1	0.03
international journal of molecular sciences	139	4.9	Q1	334	84735	nature medicine	6581	58.7	Q1	478	25123	new england journal of medicine	8116	96.3	Q1	0
cancer immunology immunotherapy	124	4.6	Q2	389	98721	new england journal of medicine	6332	96.3	Q1	385	5047	plos one	7019	2.9	Q1	0.01
acs applied materials & interfaces	116	8.5	Q1	291	34120	journal of clinical investigation	5588	13.3	Q1	511	70621	**frontiers in immunology**	6928	5.7	Q1	**0.22**
plos one	113	2.9	Q1	226	62946	**frontiers in immunology**	5500	5.7	Q1	**938**	**307807**	nature reviews cancer	6754	72.5	Q1	0
cancer letters	112	9.1	Q1	465	88841	cancer cell	5479	48.8	Q1	410	54459	cancer cell	6727	48.8	Q1	0
cancer immunology research	103	8.1	Q1	474	111587	acs nano	5296	15.8	Q1	321	33201	nature medicine	6411	58.7	Q1	0

TLSCA, Total link strength of citation analysis; TLSBCA, Total link strength of bibliographic coupling analysis; PNAS, proceedings of the national academy of sciences of the united states of america.

Bold values represent the top three values in each item.

The citation analysis primarily examines the reciprocal citation relationships among these 1,612 journals. A citation network was constructed based on the top 15 most productive journals (**Figure 3B**). Within this network, Frontiers in Immunology exhibited the strongest mutual citation relationships with Oncoimmunology (links = 37), the Journal for ImmunoTherapy of Cancer (links = 34), and Cancer Research (links = 28) (**Figure 3B**). The three journals with the highest total link strength in the citation analysis were Nature Communications, Frontiers in Immunology, and the Journal for ImmunoTherapy of Cancer (**Table 2**).

The bibliographic coupling analysis investigates the citation of shared references across the 1,612 journals. A bibliographic coupling network was established based on the top 15 most productive journals (**Figure 3C**). Among these journals, Frontiers in Immunology demonstrated the strongest bibliographic coupling relationships with Nature Communications (links = 10,333), the Journal for ImmunoTherapy of Cancer (links = 8,905), and Frontiers in Oncology (links = 8,861) (**Figure 3C**). The three journals exhibiting the highest total link strength in the bibliographic coupling analysis were Frontiers in Immunology, Nature Communications, and the Journal for ImmunoTherapy of Cancer (**Table 2**).

#### 3.2.3 Most cited journals

Among these 1,612 journals, Nature was identified as the most cited, followed by Nature Communications and Cancer Research (**Table 2**). A citation network was constructed based on the top 15 most cited journals (**Figure 3D**). Within this network, Nature exhibited the strongest mutual citation relationships with Nature Communications (links = 92), the Journal for ImmunoTherapy of Cancer (links = 56), and Frontiers in Immunology (links = 48) (**Figure 3D**). The three journals with the highest total link strength in the citation analysis were Nature, Nature Communications, and Frontiers in Immunology (**Table 2**).

Additionally, a bibliographic coupling network was established based on the top 15 most cited journals (**Figure 3E**). Among these journals, Nature demonstrated the strongest bibliographic coupling relationships with Nature Communications (links = 2,944), Frontiers in Immunology (links = 2,474), and the Journal for ImmunoTherapy of Cancer (links = 2,116) (**Figure 3E**). The three journals exhibiting the highest total link strength in the bibliographic coupling analysis were Frontiers in Immunology, Nature Communications, and the Journal for ImmunoTherapy of Cancer (**Table 2**).

To summarize, Frontiers in Immunology, Nature Communications, the Journal for ImmunoTherapy of Cancer, and Nature are identified as the four most influential journals on tumor immune escape.

#### 3.2.4 Most co-cited journals

A total of 331,872 cited references related to tumor immune escape were published across 14,355 different co-cited journals. Among these 14,355 journals, Nature was the most co-cited journal, followed by Cancer Research, Proceedings of the National Academy of Sciences of the United States of America, Cell, and Clinical Cancer Research (**Table 2**). Within the top 15 most co-cited journals, four journals exhibited a centrality score exceeding 0.15, as determined by CiteSpace software: Cancer Research, Frontiers in Immunology, Nature, and Clinical Cancer Research (**Table 2**).

#### 3.2.5 Dual-map overlay analysis of journals

The application of CiteSpace software enabled the generation of a dual-map overlay of academic journals, illustrating the relationships between citing and cited journals. The left side of the map represents the citing journals, while the right side depicts the cited journals. As illustrated in **Figure 3F**, three primary pathways are evident: two represented in orange and one in green. The two orange pathways signify that journals within the domain of Molecular/Biology/Immunology frequently reference articles from journals in the fields of Molecular/Biology/Genetics (z-score = 9.95) and Health/Nursing/Medicine (z-score = 1.99). Conversely, the green pathway indicates that journals within the domain of Medicine/Medical/Clinical consistently cite articles from journals in the field of Molecular/Biology/Genetics (z-score = 2.88).

### 3.3 Analysis of authors

#### 3.3.1 Most productive authors

A total of 71,684 authors participated in research on tumor immune escape. Among these contributors, Zhang Wei emerged as the most prolific author, followed by Wang Wei and Zhang Yan. Collectively, the top ten authors produced 443 articles, which represent 3.98% of the total 11,128 articles published (**Table 3**).

**Table 3:** Top ten authors in tumor immune escape research.

Top ten most productive authors					Top ten most cited authors					Top ten most co-cited authors		
Authors	Counts	TLSCOA	TLSCA	TLSBCA	Authors	Citations	TLSCOA	TLSCA	TLSBCA	Authors	Citations	Centrality
**zhang wei**	64	**602**	1541	**445352**	**freeman gordon j**	7270	**165**	**5626**	**296544**	**hanahan d**	907	**0.14**
**wang wei**	58	**544**	**2116**	**469971**	**jiang peng**	4176	**124**	**3621**	**161677**	**topalian sl**	832	**0.29**
zhang yan	44	370	1686	309099	**liu x shirley**	4135	123	**3632**	153854	**zhang y**	737	**0.15**
liu yang	44	418	1033	291712	angelova mihaela	3967	51	2118	78598	siegel rl	724	0.01
zhang yu	41	394	1227	246326	armand philippe	3858	92	1848	62483	sharma p	656	0.09
**wang yan**	40	**448**	**2621**	**318311**	fu jingxin	3800	82	3158	109306	wang y	605	0.02
zhang jing	39	375	1747	287097	charoentong pornpimol	3739	59	1939	113348	liu y	597	0.01
**li jing**	38	370	**1939**	267534	trajanoski zlatko	3628	38	2003	120381	wang j	593	0.01
wang jing	38	393	843	271796	hackl hubert	3628	35	1892	89767	**zhang l**	588	**0.17**
wang ying	37	353	894	240400	**wucherpfennig kai w**	3565	**124**	3111	**155417**	parkin dm	576	0.04

TLSCOA, Total link strength of co-authorship analysis; TLSCA, Total link strength of citation analysis; TLSBCA, Total link strength of bibliographic coupling analysis.

Bold values represent the top three values in each item.

**Figure 4:**
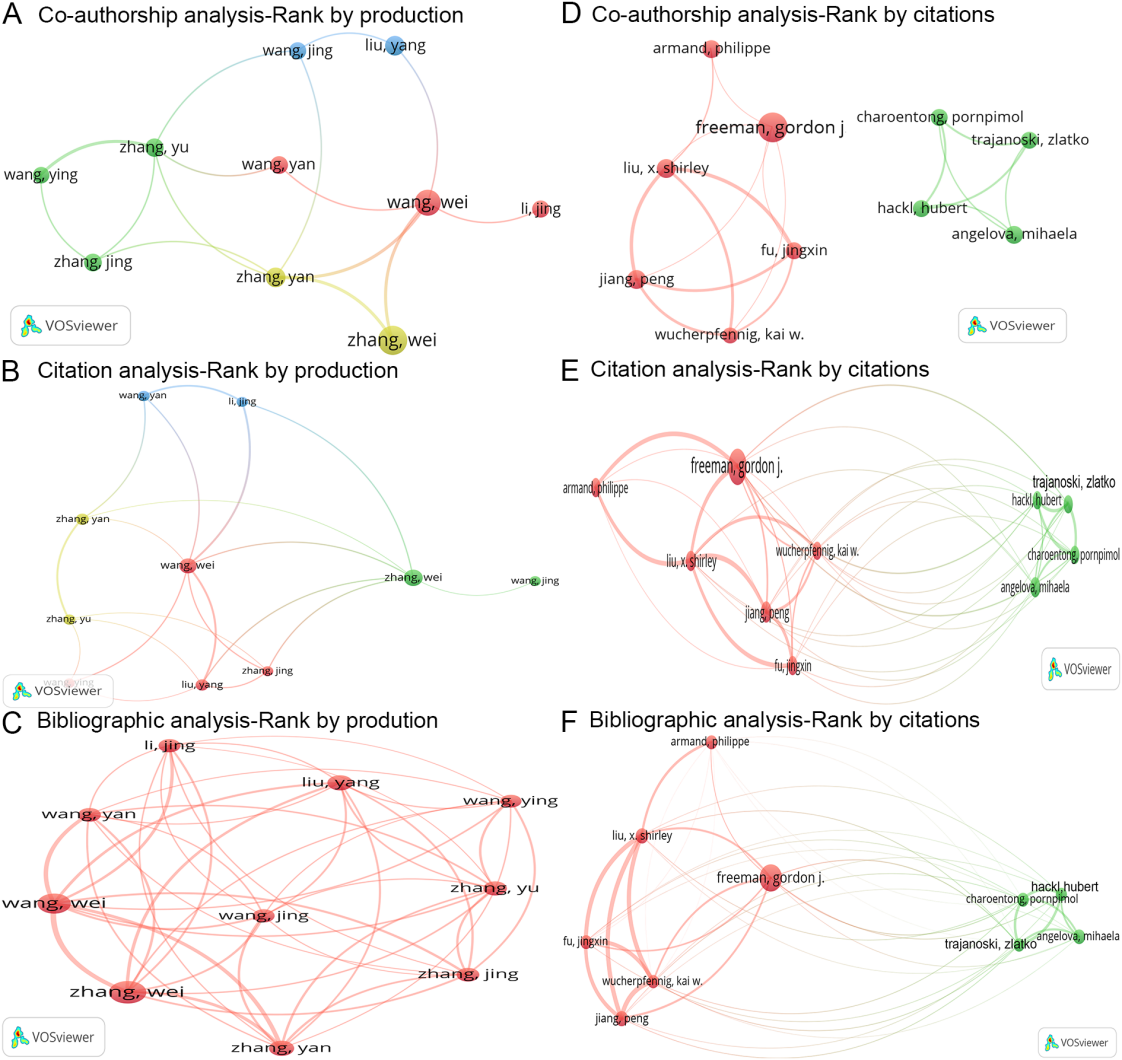
Analysis of authors. **(A-C)**. Three network maps illustrating the co-authorship, citation, and bibliographic coupling analyses of the ten most productive authors, respectively. Node size reflects the number of articles, line width indicates interaction strength, and colors represent different clusters. **(D-F)**. Three network maps depicting the co-authorship, citation, and bibliographic coupling analyses of the ten most cited authors, respectively. Node size indicates citation frequency, line width shows interaction strength, and colors denote clusters.

The co-authorship analysis focuses on the interrelationships among the 71,684 authors by quantifying the number of articles they have co-authored. A co-authorship network was developed based on the ten most productive authors (**Figure 4A**). In this network, Zhang Wei displayed co-authorships with Wang Wei (links = 2) and Zhang Yan (links = 2) (**Figure 4A**). The three authors with the highest total link strength in the co-authorship analysis were Zhang Wei, Wang Wei, and Wang Yan (**Table 3**).

The citation analysis examines the reciprocal citation relationships among the 71,684 authors. A citation network was constructed based on the top ten most productive authors (**Figure 4B**). Among these authors, Zhang Wei exhibited the strongest mutual citation relationships with Wang Wei (links = 2), Zhang Jing (links = 2), Liu Yang (links = 2), and Li Jing (links = 2) (**Figure 4B**). The three authors with the highest total link strength in the citation analysis were Wang Yan, Wang Wei, and Li Jing (**Table 3**).

The bibliographic coupling analysis investigates the citation of shared references among the 71,684 authors. A bibliographic coupling network was established based on the top ten most productive authors (**Figure 4C**). Among these authors, Zhang Wei demonstrated the strongest bibliographic coupling relationships with Wang Wei (links = 367), Zhang Yan (links = 283), and Liu Yang (links = 216) (**Figure 4C**). The three authors with the highest total link strength in the bibliographic coupling analysis were Wang Wei, Zhang Wei, and Wang Yan (**Table 3**).

Overall, the four most influential authors in the field of tumor immune escape are Wang Wei, Wang Yan, Zhang Wei, and Li Jing.

#### 3.3.2 Most cited authors

Among these 71,684 authors, Freeman Gordon J was identified as the most cited, followed by Jiang Peng and Liu X Shirley (**Table 3**). A co-authorship network was developed for the top ten most cited authors (**Figure 4D**). In this network, Freeman Gordon J displayed co-authorships with Jiang Peng (link = 1), Liu X Shirley (link = 1), Armand Philippe (link = 1), Fu Jingxin (link = 1), and Wucherpfennig Kai W (link = 1) (**Figure 4D**). The three authors with the highest total link strength in the co-authorship analysis were Freeman Gordon J, Jiang Peng, and Wucherpfennig Kai W (**Table 3**).

Additionally, a citation network was constructed for the top ten most cited authors (**Figure 4E**). Among these authors, Freeman Gordon J exhibited the strongest mutual citation relationships with Liu X Shirley (links = 8), Armand Philippe (links = 7), and Jiang Peng (links = 5) (**Figure 4E**). The three authors with the highest total link strength in the citation analysis were Freeman Gordon J, Liu X Shirley, and Jiang Peng (**Table 3**).

Furthermore, a bibliographic coupling network was established for the top ten most cited authors (**Figure 4F**). Among these authors, Freeman Gordon J demonstrated the strongest bibliographic coupling relationships with Jiang Peng (links = 167), Wucherpfennig Kai W (links = 162), and Liu X Shirley (links = 159) (**Figure 4F**). The three authors with the highest total link strength in the bibliographic coupling analysis were Freeman Gordon J, Jiang Peng, and Wucherpfennig Kai W (**Table 3**).

All in all, the four most influential authors in the domain of tumor immune escape are Freeman Gordon J, Jiang Peng, Liu X Shirley, and Wucherpfennig Kai W.

#### 3.3.3 Most co-cited authors

This study analyzed 174,657 co-cited authors from a total of 331,872 references. Among these authors, Hanahan D was the most co-cited, followed by Topalian SL and Zhang Y (**Table 3**). Notably, four authors among the top ten most co-cited individuals achieved a centrality score exceeding 0.1, as determined by CiteSpace software: Topalian SL, Zhang L, Zhang Y, and Hanahan D (**Table 3**).

### 3.4 Analysis of institutions

#### 3.4.1 Most productive institutions

The investigation into tumor immune escape encompassed a total of 9,254 institutions. The leading three institutions in terms of article publication were Fudan University, Sun Yat-sen University, and Shanghai Jiao Tong University. Collectively, the top ten most productive institutions contributed 2,424 articles, representing 21.78% of the overall 11,128 articles published (**Table 4**).

**Table 4:** Top ten institutions in tumor immune escape research.

Top ten most productive institutions					Top ten most cited institutions				
Institutions	Counts	TLSCOA	TLSCA	TLSBCA	Institutions	Citations	TLSCOA	TLSCA	TLSBCA
**fudan univ**	329	659	**4375**	**1037830**	**harvard med sch**	22098	**1404**	**7740**	**1168270**
**sun yat-sen univ**	303	694	4269	**976755**	**dana farber canc inst**	19844	854	**6737**	690854
shanghai jiao tong univ	301	684	3789	866507	**univ texas md anderson canc ctr**	14986	**871**	**5047**	**758805**
**chinese acad sci**	284	**936**	3860	798776	**chinese acad sci**	12394	**936**	3860	**798776**
**harvard med sch**	261	**1404**	**7740**	**1168270**	mem sloan kettering canc ctr	12100	565	3153	439150
zhejiang univ	213	461	2763	520717	massachusetts gen hosp	11739	680	3943	505605
sichuan univ	203	349	1888	381277	brigham & womens hosp	11498	573	3938	427526
**univ texas md anderson canc ctr**	199	**871**	**5047**	758805	harvard univ	11446	283	2616	212150
nanjing med univ	170	353	1925	416589	univ penn	11278	347	2554	317396
zhengzhou univ	161	343	1568	391421	univ calif los angeles	11037	316	3250	248314

univ, university; chinese acad sci, chinese academy of sciences; harvard med sch, Harvard Medical School; univ texas md anderson canc ctr, University of Texas MD Anderson Cancer Center; nanjing med univ, Nanjing Medical University; dana farber canc inst, Dana-Farber Cancer Institute; mem sloan kettering canc ctr, Memorial Sloan Kettering Cancer Center; massachusetts gen hosp, Massachusetts General Hospital; brigham & womens hosp, Brigham and Women’s Hospital; univ penn, University of Pennsylvania; univ calif los angeles, University of California, Los Angeles; TLSCOA, Total link strength of co-authorship analysis; TLSCA, Total link strength of citation analysis; TLSBCA, Total link strength of bibliographic coupling analysis.

Bold values represent the top three values in each item.

**Figure 5:**
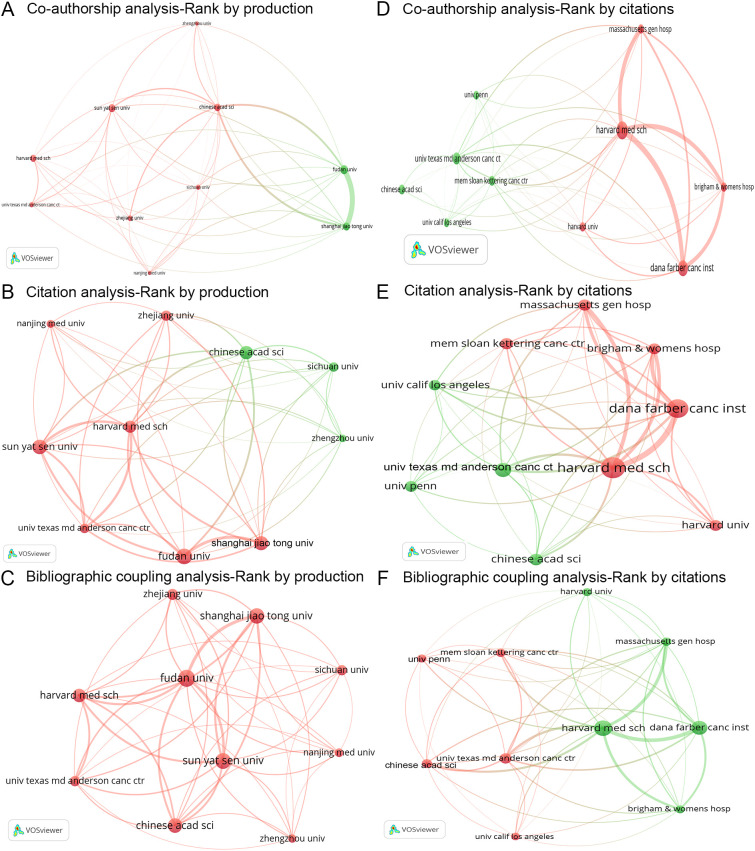
Analysis of institutions. **(A-C)** Three network maps illustrating the co-authorship, citation, and bibliographic coupling analyses of the ten most productive institutions, respectively. Node size reflects the number of articles, line width indicates interaction strength, and colors represent different clusters. **(D-F)** Three network maps depicting the co-authorship, citation, and bibliographic coupling analyses of the ten most cited institutions, respectively. Node size indicates citation frequency, line width shows interaction strength, and colors denote clusters. univ, university; chinese acad sci, chinese academy of sciences; harvard med sch, Harvard Medical School; univ texas md anderson canc ctr, University of Texas MD Anderson Cancer Center; nanjing med univ, Nanjing Medical University; dana farber canc inst, Dana-Farber Cancer Institute; mem sloan kettering canc ctr, Memorial Sloan Kettering Cancer Center; massachusetts gen hosp, Massachusetts General Hospital; brigham & womens hosp, Brigham and Women’s Hospital; univ penn, University of Pennsylvania; univ calif los angeles, University of California, Los Angeles.

The co-authorship analysis primarily focuses on the interrelationships among these 9,254 institutions by quantifying the number of collaboratively authored articles. A co-authorship network was developed based on the top ten most productive institutions (**Figure 5A**). In this network, Fudan University displayed the strongest co-authorship ties with Shanghai Jiao Tong University (links = 77), the Chinese Academy of Sciences (links = 25), Sun Yat-sen University (links = 7), and Sichuan University (links = 7) (**Figure 5A**). The three institutions with the highest total link strength in the co-authorship analysis were Harvard Medical School, the Chinese Academy of Sciences, and the University of Texas MD Anderson Cancer Center (**Table 4**).

The citation analysis primarily examines the reciprocal citation relationships among the 9,254 institutions. A citation network was constructed based on the top ten most productive institutions (**Figure 5B**). Among these institutions, Fudan University exhibited the strongest mutual citation relationships with Shanghai Jiao Tong University (links = 101), Sun Yat-sen University (links = 72), Harvard Medical School (links = 64), and the Chinese Academy of Sciences (links = 64) (**Figure 5B**). The three institutions with the highest total link strength in the citation analysis were Harvard Medical School, the University of Texas MD Anderson Cancer Center, and Fudan University (**Table 4**).

The bibliographic coupling analysis investigates the citation of shared references across the 9,254 institutions. A bibliographic coupling network was established based on the top ten most productive institutions (**Figure 5C**). Among these institutions, Fudan University demonstrated the strongest bibliographic coupling relationships with Shanghai Jiao Tong University (links = 16,199), Sun Yat-sen University (links = 13,499), and Harvard Medical School (links = 11,401) (**Figure 5C**). The three institutions with the highest total link strength in the bibliographic coupling analysis were Harvard Medical School, Fudan University, and Sun Yat-sen University (**Table 4**).

#### 3.4.2 Most cited institutions

Among the 9,254 institutions, Harvard Medical School emerged as the most frequently cited, followed by Dana-Farber Cancer Institute and the University of Texas MD Anderson Cancer Center (**Table 4**). A co-authorship network was developed based on the top ten most cited institutions (**Figure 5D**). In this network, Harvard Medical School displayed co-authorships with Dana-Farber Cancer Institute (links = 79), Massachusetts General Hospital (links = 71), and Brigham and Women’s Hospital (links = 57) (**Figure 5D**). The three institutions with the highest total link strength in the co-authorship analysis were Harvard Medical School, the Chinese Academy of Sciences, and the University of Texas MD Anderson Cancer Center (**Table 4**).

Additionally, a citation network was constructed based on the top ten most cited institutions (**Figure 5E**). Among these institutions, Harvard Medical School exhibited the strongest mutual citation relationships with Dana-Farber Cancer Institute (links = 182), Massachusetts General Hospital (links = 115), and Brigham and Women’s Hospital (links = 100) (**Figure 5E**). The three institutions with the highest total link strength in the citation analysis were Harvard Medical School, Dana-Farber Cancer Institute, and the University of Texas MD Anderson Cancer Center (**Table 4**).

Furthermore, a bibliographic coupling network was established based on the top ten most cited institutions (**Figure 5F**). Among these institutions, Harvard Medical School demonstrated the strongest bibliographic coupling relationships with Dana-Farber Cancer Institute (links = 15,864), Massachusetts General Hospital (links = 12,362), and the University of Texas MD Anderson Cancer Center (links = 10,269) (**Figure 5F**). The three institutions with the highest total link strength in the bibliographic coupling analysis were Harvard Medical School, the Chinese Academy of Sciences, and the University of Texas MD Anderson Cancer Center (**Table 4**).

In conclusion, the institutions identified as the most influential in the field of tumor immune escape include Harvard Medical School, Fudan University, Sun Yat-sen University, the Chinese Academy of Sciences, the University of Texas MD Anderson Cancer Center, and the Dana-Farber Cancer Institute.

### 3.5 Analysis of countries

#### 3.5.1 Most productive countries

The investigation into tumor immune escape encompassed a total of 121 countries. The leading contributors to the literature in this field were China, the USA, and Germany. Collectively, these three countries produced 8,901 articles, which represent 79.99% of the total 11,128 articles published (**Table 5**).

**Table 5:** Top ten countries in tumor immune escape research.

Top ten most productive countries					Top ten most cited countries				
Countries	Counts	TLSCOA	TLSCA	TLSBCA	Countries	Citations	TLSCOA	TLSCA	TLSBCA
**peoples r china**	5045	**1168**	**12654**	**3069479**	**usa**	164201	**2444**	**14878**	**2821349**
**usa**	2984	**2444**	**14878**	**2821349**	**peoples r china**	123998	**1168**	**12654**	**3069479**
**germany**	872	**1022**	**4330**	**861392**	**germany**	36710	**1022**	**4330**	**861392**
japan	526	348	2207	568117	england	20757	823	3071	574719
italy	514	611	1914	463478	japan	18344	348	2207	568117
england	430	823	3071	574719	france	17372	547	2106	414899
france	430	547	2106	414899	italy	17160	611	1914	463478
south korea	332	175	1525	337718	spain	13293	420	1934	339470
canada	300	405	1811	373048	canada	12929	405	1811	373048
australia	292	393	1970	330034	switzerland	12902	465	1491	303849

peoples r china, The People’s Republic of China; usa, The United States of America; TLSCOA, Total link strength of co-authorship analysis; TLSCA, Total link strength of citation analysis; TLSBCA, Total link strength of bibliographic coupling analysis.

Bold values represent the top three values in each item.

**Figure 6:**
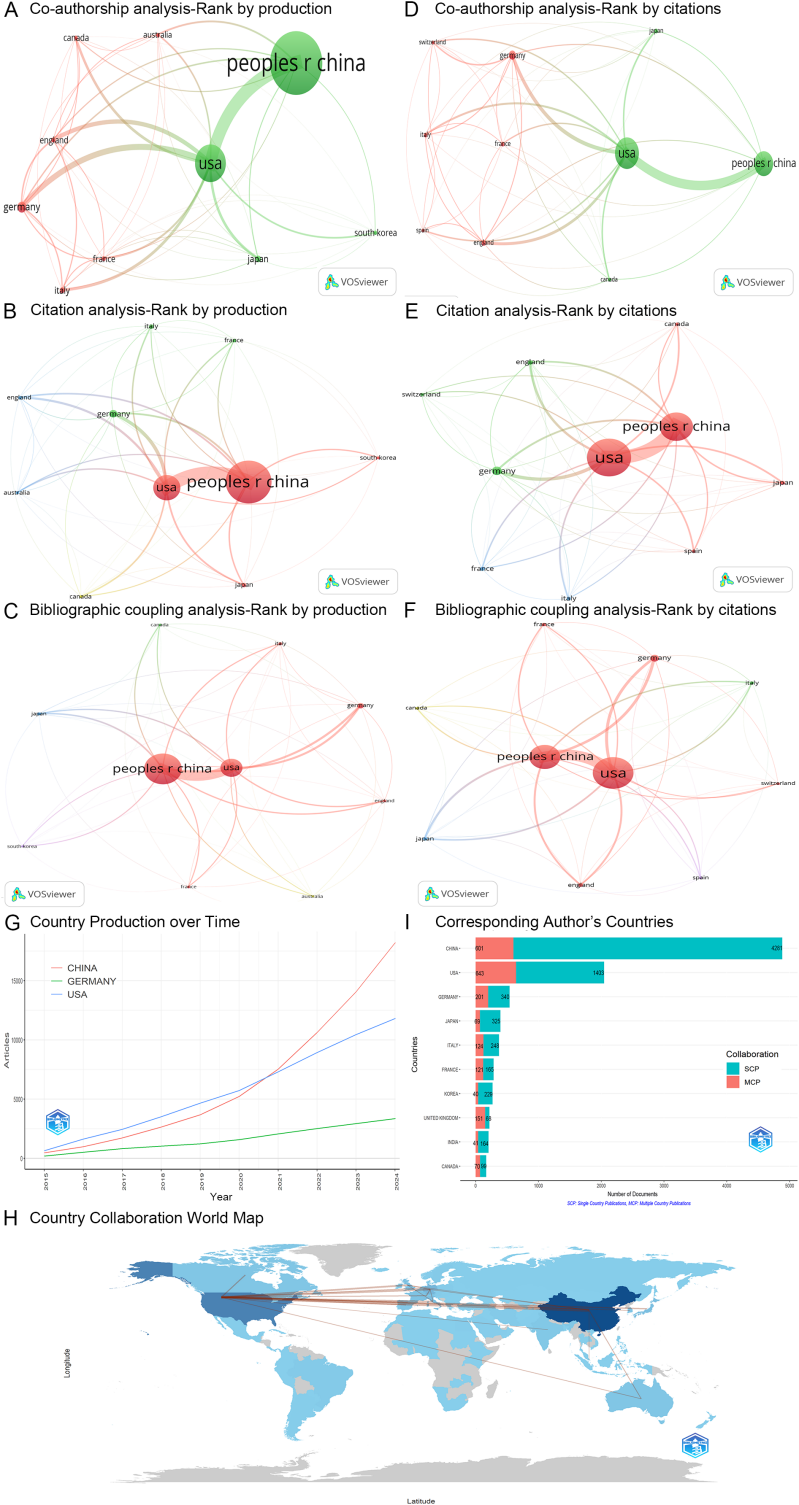
Analysis of countries. **(A-C)** Three network maps illustrating the co-authorship, citation, and bibliographic coupling analyses of the ten most productive countries, respectively. Node size reflects the number of articles, line width indicates interaction strength, and colors represent different clusters. **(D-F)** Three network maps depicting the co-authorship, citation, and bibliographic coupling analyses of the ten most cited countries, respectively. Node size indicates citation frequency, line width shows interaction strength, and colors denote clusters. **(G)** Annual production levels for the USA, China, and Germany. **(H)** A collaboration map among countries, where the width of the red lines indicates the degree of cooperation between countries. Different shades of blue represent the number of articles published by each country, with darker shades indicating a higher volume of publications. **(I)** A bar chart presenting the statistical analysis of the countries of corresponding authors for 11,128 articles. peoples r china, The People’s Republic of China; usa, The United States of America; MCP, multiple country publications; SCP, single country publications.

The co-authorship analysis focuses on the collaborative relationships among these 121 countries by quantifying the number of articles co-authored. A co-authorship network was developed based on the ten most productive countries (**Figure 6A**). In this network, China demonstrated the strongest co-authorship ties with the USA (links = 537), Australia (links = 69), and Germany (links = 58) (**Figure 6A**). The three countries with the highest total link strength in the co-authorship analysis were the USA, China, and Germany (**Table 5**).

The citation analysis primarily examines the reciprocal citation relationships among the 121 countries. A citation network was constructed based on the ten most productive countries (**Figure 6B**). Among these countries, China exhibited the strongest mutual citation relationships with the USA (links = 4,974), Germany (links = 670), and England (links = 624) (**Figure 6B**). The three countries with the highest total link strength in the citation analysis were again the USA, China, and Germany (**Table 5**).

The bibliographic coupling analysis investigates the citation of shared references across 121 countries. A bibliographic coupling network was established based on the ten most productive countries (**Figure 6C**). Among these countries, China demonstrated the strongest bibliographic coupling relationships with the USA (links = 979,591), Germany (links = 219,059), and Japan (links = 161,888) (**Figure 6C**). The three countries with the highest total link strength in the bibliographic coupling analysis were China, the USA, and Germany (**Table 5**).

#### 3.5.2 Most cited countries

Among the 121 countries analyzed, the USA emerged as the most frequently cited, followed by China and Germany (**Table 5**). A co-authorship network was developed based on the ten most cited countries (**Figure 6D**). In this network, the USA displayed co-authorships with China (links = 537), Germany (links = 225), and England (links = 164) (**Figure 6D**). The three countries with the highest total link strength in the co-authorship analysis were the USA, China, and Germany (**Table 5**).

Additionally, a citation network was constructed based on the ten most cited countries (**Figure 6E**). Among these countries, the USA exhibited the strongest mutual citation relationships with China (links = 4,974), Germany (links = 1,226), and England (links = 806) (**Figure 6E**). The three countries with the highest total link strength in the citation analysis were, once again, the USA, China, and Germany (**Table 5**).

Furthermore, a bibliographic coupling network was established based on the ten most cited countries (**Figure 6F**). Among these countries, the USA demonstrated the strongest bibliographic coupling relationships with China (links = 979,591), Germany (links = 215,195), and England (links = 141,107) (**Figure 6F**). The three countries with the highest total link strength in the bibliographic coupling analysis were China, the USA, and Germany (**Table 5**).

#### 3.5.3 Countries’ production over time

As mentioned above, the USA, China, and Germany represented the three most prominent countries in the domain of tumor immune escape research. **Figure 6G** presents a graphical representation of the annual production data for these countries, demonstrating a consistent upward trend in their respective production levels over the years. Notably, since 2021, China’s production has surpassed that of the USA.

#### 3.5.4 Country collaboration map

The USA exhibited the most significant degree of collaboration with other countries worldwide regarding tumor immune escape research. Notably, the USA maintained the strongest international partnerships with China, followed by Germany and the United Kingdom (**Figure 6H**).

#### 3.5.5 Corresponding author’s countries

According to multiple country publications (MCP), the USA (n = 643) was identified as the predominant contributor, followed by China (n = 601) and Germany (n = 201). Conversely, according to single country publications (SCP), China (n = 4281) emerged as the leading contributor, with the USA (n = 1403) and Germany (n = 340) following in rank (**Figure 6I**).

In conclusion, the USA, China, and Germany are recognized as the three most significant countries in the field of tumor immune escape research. Notably, the USA and China are positioned within the same analytical cluster, indicating a stronger correlation between their contributions (**Figures 6A–F**).

### 3.6 Analysis of keywords

#### 3.6.1 Co-occurrence analysis of author keywords

**Figure 7:**
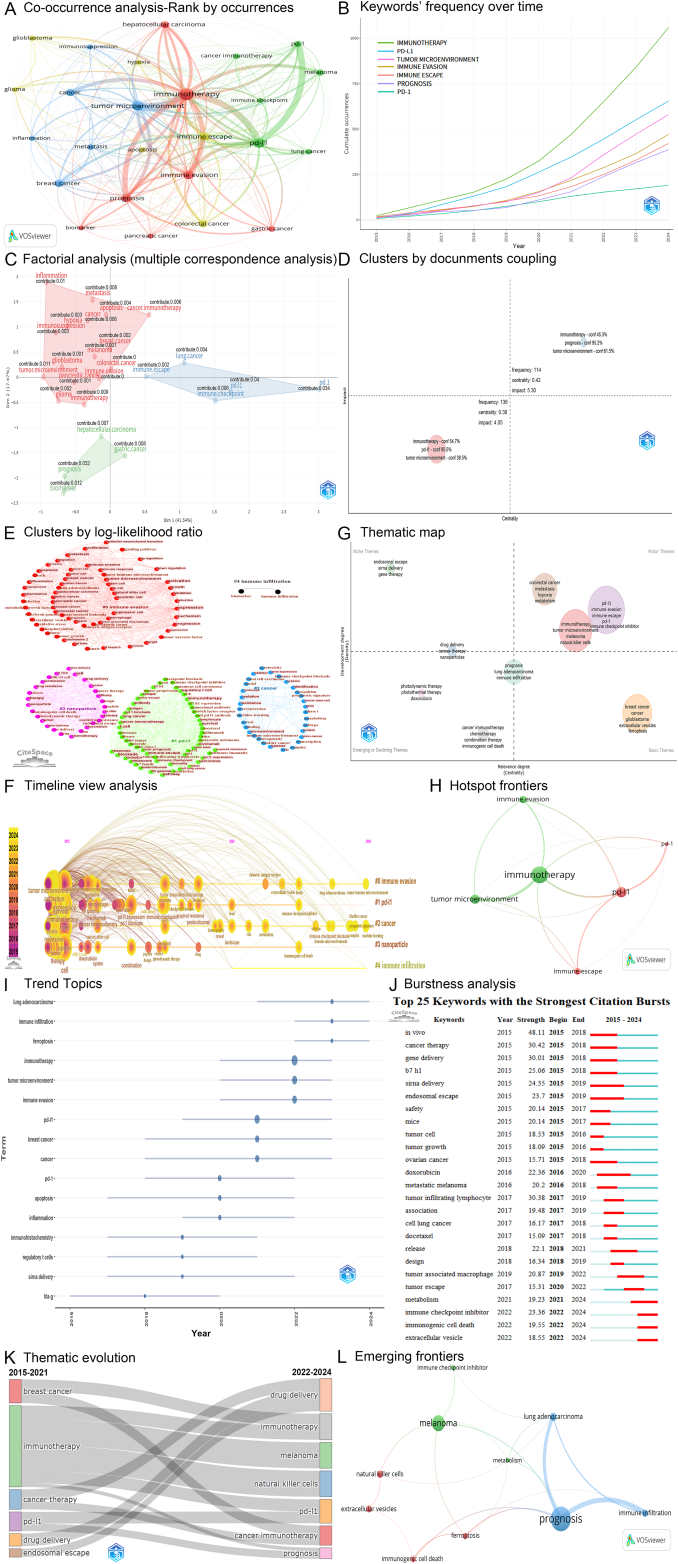
Analysis of keywords. **(A)** The network map illustrates the co-occurrence analysis of the top 25 most frequently occurring author keywords. Node size represents frequency of occurrence, line width indicates interaction strength, and colors represent different clusters. **(B)** The cumulative occurrence plot of author keywords over time. **(C)** The factorial analysis plot for the top 25 most frequently occurring author keywords. **(D)** The document coupling cluster plot based on the top 250 most frequently occurring author keywords. **(E)** The cluster plot generated by CiteSpace utilizing the log-likelihood ratio method. **(F)** The timeline view analysis corresponding to **(E)**. **(G)** The thematic map of the top 250 most frequently occurring author keywords. **(H)** The co-occurrence network map of six hotspot frontiers. **(I)** The trend topics plot for the top 250 most frequently occurring author keywords. **(J)** The top 25 keywords exhibiting the strongest citation bursts. **(K)** The thematic evolution map of author keywords with a minimum frequency exceeding 50. **(L)** The co-occurrence network map of ten emerging frontiers. pd-l1, programmed cell death ligand 1; pd-1, programmed death receptor 1.

The VOSviewer software was used, resulting in 16,465 author keywords. Co-occurrence analysis of author keywords reveals research frontiers by analyzing the co-occurrence relationships among these 16,465 keywords across the 11,128 articles. The five most frequently occurring author keywords identified in this analysis were immunotherapy, programmed cell death ligand 1 (PD-L1), tumor microenvironment, immune evasion, and immune escape (**Figure 7A**, **Table 6**). We classified the top 25 most frequent author keywords into five categories, namely immune checkpoints, immunotherapy, tumor microenvironment, tumors, and others. The immune checkpoints category includes immune checkpoint, PD-L1, and PD-1. The immunotherapy category encompasses both immunotherapy and cancer immunotherapy. The tumor microenvironment category consists of tumor microenvironment and immunosuppression. The tumors category includes cancer, breast cancer, hepatocellular carcinoma, colorectal cancer, melanoma, lung cancer, gastric cancer, glioblastoma, pancreatic cancer, and glioma. The others category comprises immune evasion, immune escape, prognosis, metastasis, apoptosis, inflammation, biomarker, and hypoxia.

**Table 6:** Top 25 most frequent author keywords in tumor immune escape research.

Top 25 most frequent author keywords	Occurrences	Total link strength of co-occurrence analysis
**immunotherapy**	1059	**4890**
**pd-l1**	653	**2925**
**tumor microenvironment**	582	**2679**
**immune evasion**	471	**2300**
**immune escape**	421	**1895**
prognosis	387	1653
breast cancer	326	1485
cancer	306	1584
hepatocellular carcinoma	275	1163
colorectal cancer	268	1157
melanoma	217	1002
pd-1	191	947
metastasis	170	873
cancer immunotherapy	167	744
lung cancer	156	677
gastric cancer	156	641
glioblastoma	146	680
apoptosis	143	694
pancreatic cancer	124	554
glioma	120	543
immune checkpoint	119	598
inflammation	114	589
biomarker	111	500
hypoxia	110	552
immunosuppression	110	530

pd-l1: programmed cell death ligand 1; pd-1: programmed death receptor 1.

Bold values represent the top three values in each item.

A co-occurrence network was constructed based on the 25 most frequently occurring author keywords (**Figure 7A**). Within this network, immunotherapy exhibited the strongest co-occurrence relationships with tumor microenvironment (links = 153), PD-L1 (links = 96), and prognosis (links = 74). PD-L1 showed the strongest co-occurrence relationships with immunotherapy (links = 96), programmed death receptor 1 (PD-1, links = 91), and immune escape (links = 68). Tumor microenvironment demonstrated the strongest co-occurrence relationships with immunotherapy (links = 153), prognosis (links = 49), and immune evasion (links = 44). Immune evasion displayed the strongest co-occurrence relationships with immunotherapy (links = 69), PD-L1 (links = 52), and tumor microenvironment (links = 44). Immune escape revealed the strongest co-occurrence relationships with PD-L1 (links = 68), immunotherapy (links = 39), and tumor microenvironment (links = 32) (**Figure 7A**). The five author keywords with the highest total link strength in the co-occurrence analysis were immunotherapy, PD-L1, tumor microenvironment, immune evasion, and immune escape (**Table 6**).

In summary, the key concepts identified include immunotherapy, PD-L1, tumor microenvironment, immune evasion, immune escape, prognosis, and PD-1, which emerged as the seven most significant research frontiers. **Figure 7B** illustrates the cumulative occurrence data for these seven keywords, indicating a consistent annual increase in their occurrence levels.

#### 3.6.2 Factorial analysis of author keywords

A factorial analysis was performed utilizing the multiple correspondence analysis method, focusing on the 25 most frequently occurring author keywords. This analysis resulted in the formation of three distinct clusters. The concepts of PD-L1, prognosis, and tumor microenvironment were the primary contributors to the blue, green, and red clusters, respectively (**Figure 7C**).

#### 3.6.3 Document coupling clustering analysis of author keywords

A document coupling clustering analysis was conducted, labeled by author keywords, focusing on the 250 most frequently occurring keywords. This analysis yielded two distinct clusters, with immunotherapy, PD-L1, prognosis, and tumor microenvironment being the most influential concepts within these clusters (**Figure 7D**).

#### 3.6.4 Clustering and timeline view analyses of keywords

**Figure 7E** presents a clustering analysis where each color represents a distinct cluster, numbered #0 through #4, resulting in a total of five clusters formed by the log-likelihood ratio (LLR). The modularity Q value was calculated to be 0.28, while the mean silhouette value was determined to be 0.75. The number of keywords in clusters #0, #1, #2, #3, and #4 is 62, 56, 32, 28, and 2, respectively. In **Figure 7E**, cluster #0 reflects a close relationship between immune escape and six immune-related cell types: stem cells, suppressor cells, natural killer (NK) cells, dendritic cells, macrophages, and tumor-associated macrophages. Cluster #1 highlights the close association between PD-L1 and four immune-related cell types: T cells, regulatory T cells, lymphocytes, and tumor-infiltrating lymphocytes. Five research fields related to tumor immune escape were identified through clustering analysis: immune evasion (#0), PD-L1 (#1), cancer (#2), nanoparticle (#3), and immune infiltration (#4). A timeline view analysis of these five keyword-based clusters was also conducted. As depicted in **Figure 7F**, each line represents a cluster, numbered #0 through #4. Immune evasion (#0), PD-L1 (#1), cancer (#2), and nanoparticle (#3) have been the primary research focuses in tumor immune escape since 2015. The clusters of immune evasion (#0) and cancer (#2) have persisted from 2015 to 2024, indicating their significance as important topics. The cluster of immune infiltration (#4) has remained relevant from 2020 to 2024, highlighting its significance as an emerging frontier.

#### 3.6.5 Hotspot frontiers in tumor immune escape research

The research frontiers encompass both hotspot and emerging frontiers. As mentioned above, the eight most significant keywords identified were immunotherapy, PD-L1, tumor microenvironment, immune evasion, immune escape, prognosis, PD-1, and cancer. **Figure 7G** illustrates a thematic map that depicts the development and relevance of these keywords. Immunotherapy, tumor microenvironment, PD-L1, immune evasion, immune escape, and PD-1 are situated in the Motor Themes quadrant, indicating that these keywords represent core themes characterized by high centrality and density. Conversely, prognosis is located in the Emerging and Basic Themes quadrant, signifying its status as an emerging theme characterized by medium centrality and density. Additionally, cancer is positioned in the Basic Themes quadrant, suggesting it represents a foundational theme characterized by high centrality and low density.

Therefore, immunotherapy, tumor microenvironment, PD-L1, immune evasion, immune escape, and PD-1 are identified as the hotspot frontiers in tumor immune escape research. The VOSviewer software was employed to visualize these six prominent topics (**Figure 7H**).

#### 3.6.6 Trend topics analysis of author keywords

A trend topics analysis was conducted, visualizing the annual evolution of primary keywords using Biblioshiny based on time series, with the aim of identifying research trends and emerging frontiers in tumor immune escape research. Specific parameters included a designated timeframe from 2015 to 2024, a minimum word frequency threshold of 50, and a maximum of three words per year. **Figure 7I** presents each topic as a line, with the span representing duration and the circle denoting the most prevalent year for that topic. The size of the circle indicates the frequency of the topic’s occurrence. As illustrated in the top-left corner of **Figure 7I**, several new keywords have gained prominence over the past three years, highlighting lung adenocarcinoma, immune infiltration, and ferroptosis as emerging frontiers in tumor immune escape research.

#### 3.6.7 Citation burstness analysis of keywords

A citation burst analysis was performed on keywords, identifying the top 25 keywords with the highest strength. **Figure 7J** illustrates the citation bursts of these keywords, with time intervals marked in blue and the durations of the citation bursts indicated in red. Notably, the citation burst periods for terms in the bottom right-hand corner of **Figure 7J**, such as metabolism (2021 – 2024), immune checkpoint inhibitors (2022 – 2024), immunogenic cell death (2022 – 2024), and extracellular vesicles (2022 – 2024), are sustained in 2024, indicating a significant surge in scholarly interest within specific research fields. In particular, immune checkpoint inhibitors exhibit the strongest citation bursts (strength = 23.36), further underscoring their prevalence and significance in the current research landscape. Collectively, these findings identify metabolism, immune checkpoint inhibitors, immunogenic cell death, and extracellular vesicles as emerging frontiers in tumor immune escape.

#### 3.6.8 Thematic evolution analysis of author keywords

**Figure 7K** presents a thematic evolution map that illustrates the development and interconnections of keywords across different periods. The keywords that have remained prevalent and enduring from 2015 to 2024 include immunotherapy, PD-L1, and drug delivery. Notably, several emerging keywords, such as prognosis, melanoma, and natural killer cells, have gained significant prominence between 2022 and 2024.

#### 3.6.9 Emerging frontiers in tumor immune escape research

The VOSviewer, Biblioshiny, and CiteSpace tools were employed to visualize the primary keywords from the past decade, analyzing emerging frontiers in tumor immune escape research. As mentioned above, the ten emerging frontiers identified include lung adenocarcinoma, immune infiltration, ferroptosis, metabolism, immune checkpoint inhibitors, immunogenic cell death, extracellular vesicles, prognosis, melanoma, and natural killer cells (**Figure 7I-K**). As depicted in **Figure 7G**, immune checkpoint inhibitors, natural killer cells, melanoma, and metabolism are situated in the Motor Themes quadrant, indicating that these keywords represent core themes characterized by high centrality and density. Prognosis, lung adenocarcinoma, and immune infiltration are located in the Emerging and Basic Themes quadrant, signifying their status as emerging themes characterized by medium centrality and density. Additionally, immunogenic cell death is positioned in the Emerging Themes quadrant, suggesting it represents an emerging theme characterized by medium centrality and low density. Furthermore, ferroptosis and extracellular vesicles are found in the Basic Themes quadrant, indicating that these keywords represent emerging themes characterized by high centrality and low density. Consequently, these ten keywords are identified as the emerging frontiers in tumor immune escape research. The VOSviewer software was utilized to visualize these emerging topics (**Figure 7L**).

### 3.7 Analysis of references

#### 3.7.1 Co-citation analysis of references

**Figure 8:**
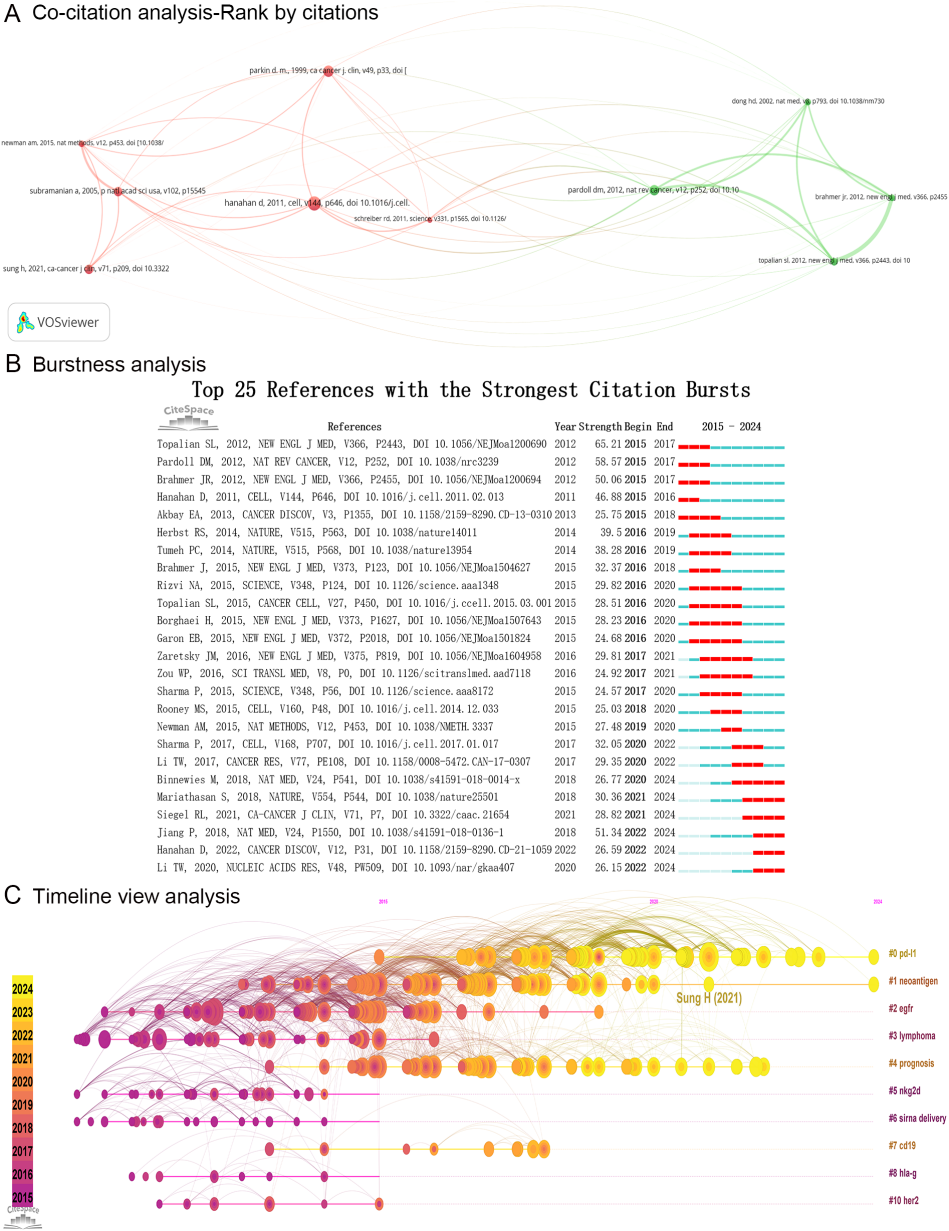
Analysis of references. **(A)** The network map illustrates the co-citation analysis of the ten most co-cited references. Node size indicates the citation frequency of references, line width indicates interaction strength, and colors represent different clusters. **(B)** The top 25 references exhibiting the strongest citation bursts. **(C)** The timeline view analysis of references generated by CiteSpace utilizing the log-likelihood ratio method. ca cancer j, CA-A Cancer Journal for Clinicians; nat rev cancer, Nature Reviews Cancer; p natl acad sci usa, Proceedings of the National Academy of Sciences of the United States of America; new engl j med, New England Journal of Medicine; nat med, Nature Medicine; nat methods, Nature Methods; cancer discov, Cancer Discovery; sci transl med, Science Translational Medicine; cancer res, Cancer Research; nucleic acids res, Nucleic Acids Research; pd-l1, Programmed cell death ligand 1; egfr, Epidermal Growth Factor Receptor; nkg2d, Natural killer group 2 member D; hla-g, Human leukocyte antigen-G; her2, Human epidermal growth factor receptor 2.

Over the past decade, a total of 331,872 references pertaining to tumor immune escape have been co-cited across 11,128 articles. The three most frequently cited references among these articles were “Hanahan D, 2011,” “Parkin DM, 1999,” and “Pardoll DM, 2012.” Each of the top ten co-cited references was cited together at least 289 times, with two references exceeding 500 citations (**Table 7**). A co-citation network was constructed focusing on the ten most co-cited references (**Figure 8A**). Within this network, “Hanahan D, 2011” exhibited the strongest co-citation relationships with “Schreiber RD, 2011” (links = 46), “Parkin DM, 1999” (links = 42), and “Subramanian A, 2005” (links = 40) (**Figure 8A**).

**Table 7:** Top ten most cited references in tumor immune escape research.

Top ten most cited references	Citations	Total link strength of co-citation analysis	First author	Year	Journal	IF (2023)	Q
**Hallmarks of Cancer: The Next Generation**	643	**44162**	hanahan d	2011	cell	45.6	Q1
Global cancer statistics	529	27878	parkin d. m	1999	ca cancer j clin	521.6	Q1
**The blockade of immune checkpoints in cancer immunotherapy**	479	**27926**	pardoll dm	2012	nat rev cancer	72.5	Q1
**Gene set enrichment analysis: A knowledge-based approach for interpreting genome-wide expression profiles**	441	**27961**	subramanian a	2005	p natl acad sci usa	9.4	Q1
Global cancer statistics 2020: GLOBOCAN estimates of incidence and mortality worldwide for 36 cancers in 185 countries	441	25431	sung h	2021	ca cancer j clin	521.6	Q1
Safety, Activity, and Immune Correlates of Anti-PD-1 Antibody in Cancer	378	20853	topalian sl	2012	new engl j med	96.3	Q1
Tumor-associated B7-H1 promotes T-cell apoptosis: A potential mechanism of immune evasion	314	16892	dong hd	2002	nat med	58.7	Q1
Robust enumeration of cell subsets from tissue expression profiles	302	17286	newman am	2015	nat methods	36.1	Q1
Safety and Activity of Anti-PD-L1 Antibody in Patients with Advanced Cancer	300	16514	brahmer jr	2012	new engl j med	96.3	Q1
Cancer Immunoediting: Integrating Immunity’s Roles in Cancer Suppression and Promotion	289	18609	schreiber rd	2011	science	44.8	Q1

ca cancer j, CA-A Cancer Journal for Clinicians; nat rev cancer, Nature Reviews Cancer; p natl acad sci usa, Proceedings of the National Academy of Sciences of the United States of America; new engl j med, New England Journal of Medicine; nat med, Nature Medicine; nat methods, Nature Methods; IF, Impact Factor; Q, Journal Citation Reports Quartile. PD-L1, programmed cell death ligand 1; PD-1, programmed death receptor 1.

Bold values represent the top three values in each item.

#### 3.7.2 Citation burstness analysis of references

A citation burst analysis was performed on the references, leading to the identification of the top 25 references exhibiting the highest burst strength. **Figure 8B** depicts the citation bursts for these references, with each bar representing a year; time intervals are marked in blue, while the durations of the citation bursts are indicated in red. The six references “Jiang P, 2018”, “Mariathasan S, 2018”, “Siegel RL, 2021”, “Binnewies M, 2018”, “Hanahan D, 2022”, and “Li TW, 2020” continuously burst in the recent three years (**Figure 8B**). Notably, “Jiang P, 2018” exhibited the strongest citation burst (strength = 51.34) and developed a computational framework known as Tumor Immune Dysfunction and Exclusion (TIDE) to predict responses to cancer immunotherapy (27). Furthermore, “Siegel RL, 2021” demonstrated a citation burst (strength = 28.82) and published cancer statistics data for 2021 (28). Additionally, “Hanahan D, 2022” showed a citation burst (strength = 26.59) and outlined the hallmarks of cancer conceptualization (29). Moreover, “Binnewies M, 2018” experienced a citation burst (strength = 26.77) and highlighted the significance of the tumor microenvironment in effective immunotherapy (30). As noted in author analyses, the tumor microenvironment has emerged as a hotspot frontier in tumor immune escape research (**Figure 7H**). On the other hand, “Mariathasan S, 2018” exhibited a citation burst (strength = 30.36) and indicated that TGF-β shaped the tumor microenvironment to inhibit anti-tumor immunity by restricting T cell immune infiltration (31). “Li TW, 2020” also demonstrated a citation burst (strength = 26.15) and improved the web platform TIMER to TIMER 2.0 for immune infiltration analysis (32). As highlighted in author analyses, immune infiltration represents an emerging frontier in tumor immune escape research (**Figure 7L**).

#### 3.7.3 Timeline view analysis of references

A timeline view analysis was conducted on these references, allowing for the identification of the most prevalent terms within each cluster, which were subsequently used as cluster labels. As illustrated in **Figure 8C**, each line corresponds to a cluster, numbered from #0 to #10, resulting in a total of eleven clusters formed through the LLR method. The modularity Q value was calculated to be 0.57, indicating a strong clustering effect and tight internal connections within the clusters. The mean silhouette value was calculated to be 0.84, suggesting that the keywords within the same cluster were highly similar and that the distinctions between different clusters were pronounced. The clusters pertaining to PD-L1 (#0) and neoantigens (#1) emerged as early as 2015 and persisted through 2024.

## 4 Discussion

This study aims to achieve a comprehensive understanding of the global distribution, knowledge systems, research hotspots, emerging frontiers, and future directions in tumor immune escape research through a medical bibliometric analysis.

### 4.1 General information and distribution

In this study, we analyzed 11,128 articles sourced from 1,612 journals, authored by 71,684 individuals affiliated with 9,254 institutions across 121 countries, published between January 1, 2015, and November 30, 2024, as indexed in the WoSCC database. The publication count rose from 613 articles in 2015 to 1,622 in 2024, indicating a 2.65-fold increase in publication volume over this period. This consistent annual growth in publications related to tumor immune escape suggests an increasing interest in this field.

Among the 11,128 articles analyzed, Jiang (2018a), Charoentong (2017), and Zaretsky (2016) are the three most influential documents on tumor immune escape, as determined by citation and bibliographic coupling analyses. The four most influential journals in this area, based on publication, citation, and bibliographic coupling analyses, are Frontiers in Immunology, Nature Communications, the Journal for ImmunoTherapy of Cancer, and Nature, all of which are classified as JCR Q1 and possess impact factors exceeding 5.

Based on the volume of publications and citations attributed to each author, institution, and country, as well as analyses of co-authorship, citation, and bibliographic coupling, the eight most influential authors in tumor immune escape research among the 71,684 authors analyzed are Wang Wei, Wang Yan, Zhang Wei, Li Jing, Freeman Gordon J, Jiang Peng, Liu X Shirley, and Wucherpfennig Kai W. Furthermore, the six most prominent institutions identified among 9,254 institutions are Harvard Medical School, Fudan University, Sun Yat-sen University, the Chinese Academy of Sciences, the University of Texas MD Anderson Cancer Center, and the Dana-Farber Cancer Institute. Additionally, the USA, China, and Germany are the three leading countries in tumor immune escape research among the 121 countries analyzed. In the realm of international collaboration in tumor immune escape research, the USA exhibits the greatest influence and has established the most extensive network of partnerships. Notably, the USA and China have formed the closest research partnership.

### 4.2 Hotspot frontiers

Eugene Garfield launched the Science Citation Index (SCI) in 1964, providing a crucial data foundation and analytical tool for scientometrics (33). The Web of Science database, derived from the Science Citation Index and currently managed by Clarivate Analytics, has become an essential resource for scientometric research, facilitating the widespread application of scientometrics (33, 34). Utilizing scientometric and citation analysis methodologies, Clarivate and the Chinese Academy of Sciences have employed big data technology to conduct quantitative analyses of scientific research frontiers. From 2014 to 2024, they published the Research Fronts™ reports for 11 consecutive years, categorizing scientific research frontiers into hotspot and emerging frontiers (35). This classification aids researchers in understanding current research trends and provides a scientific basis for policy-making, optimal allocation of scientific research resources, and strategic planning for future science and technology. Hotspot frontiers are research domains that have established a solid foundation, are currently experiencing rapid development, and are attracting significant attention. Through comprehensive analyses of keyword co-occurrence, frequency, factorial design, clustering, timeline view, and thematic mapping, we have identified potential hotspot frontiers in the field of tumor immune escape. The findings indicate that the primary research hotspot frontiers in this domain are centered on immunotherapy, the tumor microenvironment, PD-L1, and PD-1.

#### 4.2.1 Tumor immune escape represents the primary challenge in immunotherapy

Patients exhibit heterogeneity in their responses to immunotherapy (36). Not all cancer patients derive benefit from this treatment; some demonstrate no initial response, while others may develop secondary drug resistance following an initial positive response (37, 38). For patients who are initially unresponsive or insensitive to immunotherapy, in-depth research should focus on elucidating the mechanisms underlying initial immune escape (39). Understanding these mechanisms could facilitate the sensitization of these patients to immunotherapy and broaden its applications (40). Microsatellite-stable colorectal cancer is the main type of colorectal cancer. It exhibits initial immune escape, demonstrates insensitivity to immunotherapy, and shows very little clinical efficacy. This immune escape mechanism may be related to the following molecular signaling targets: downregulation of human leukocyte antigen class I molecules and upregulation of immune checkpoint molecules (39). For patients who initially respond but subsequently develop secondary drug resistance, investigating the mechanisms of secondary immune escape could yield new insights into enhancing treatment efficacy, overcoming resistance, and improving patient prognosis (37, 41). C-X-C chemokine receptor type 2 (CXCR2) antagonist AZD5069 overcame immunotherapy secondary resistance in hepatocellular carcinoma by targeting myeloid interleukin-8/CXCR2 signaling (41). Tumor immune escape represents a fundamental obstacle in the field of immunotherapy (42), necessitating ongoing investigation and research.

#### 4.2.2 Tumor immune escape and the tumor microenvironment

The tumor microenvironment (TME) constitutes a complex ecosystem surrounding tumor cells, comprising various cellular and non-cellular components (43). It fosters an immunosuppressive environment that facilitates tumor immune escape through multiple mechanisms, such as inhibitory immune cells (44), immune checkpoints (19), and cytokines (45). The TME serves as a core driving force behind tumor immune escape (44), representing a key target for overcoming immune evasion and a critical factor influencing the efficacy of immunotherapy (46). Hepatocellular carcinoma attains immune escape via the regulatory T cells of the tumor microenvironment, which play a crucial role in facilitating the progression of this malignancy. Regulatory T cells frequently exhibit high expression of PD-L1 and cytotoxic T-lymphocyte-associated protein 4 (CTLA-4), thereby promoting the immune escape of hepatocellular carcinoma cells (44). The colony-stimulating factor-1 receptor and focal adhesion kinase offer a unique opportunity to mitigate tumor immune escape and enhance the efficacy of immunotherapy and conventional cytotoxic therapy by reprogramming the tumor microenvironment (46). Immune cells are crucial cellular components of the TME. Cluster #0 in **Figure 7E** illustrates the close relationship between immune escape and six immune-related cell types. The tumor microenvironment is an important hotspot frontier in tumor immune escape research.

#### 4.2.3 PD-1 and PD-L1

Programmed death receptor 1 (PD-1) and programmed cell death ligand 1 (PD-L1) are pivotal immune checkpoint molecules (47). PD-1 is predominantly expressed on the surface of immune cells (48), whereas PD-L1 is primarily located on the surface of tumor cells and antigen-presenting cells (49). The interaction between PD-1 and PD-L1 is crucial in facilitating tumor immune escape (50). When PD-1 binds to PD-L1, it inhibits T cell activation and proliferation, resulting in immunosuppression (47). This mechanism represents a significant target for tumor immunotherapy (50). Cluster #1 in **Figure 7E** highlights the close relationship between PD-L1 and four immune-related cell types. PD-1 and PD-L1 are important hotspot frontiers in the study of tumor immune escape mechanisms. In 2014, pembrolizumab, the first PD-1 inhibitor, was approved by the United States Food and Drug Administration (FDA) (51). In 2016, atezolizumab, the first PD-L1 inhibitor, received FDA approval (52). From 2014 to 2024, monoclonal antibodies targeting PD-1 or PD-L1 received continuous approval for marketing and expanded indications (53). Over the past decade, PD-1 and PD-L1 have emerged as significant hotspots in the study of tumor immune escape. The research related to PD-1/PD-L1 has elucidated the molecular mechanisms underlying tumor immune escape, offering insights into potential solutions for overcoming drug resistance to immunotherapy.

### 4.3 Emerging frontiers

Emerging frontiers represent research fields that have recently emerged and are currently in the exploratory stage, demonstrating significant potential for innovation and development. Through comprehensive analyses of trend topics, citation burstness, and thematic evolution, we have identified the emerging frontiers in tumor immune escape research. The findings indicate that the primary emerging frontiers in this field are focused on immune checkpoint inhibitors, immune infiltration, natural killer cells, extracellular vesicles, ferroptosis, immunogenic cell death, metabolism, lung adenocarcinoma, melanoma, and prognosis.

#### 4.3.1 Immune checkpoint inhibitors

Immune checkpoint inhibitors represent a significant breakthrough in tumor treatment (54). In 2011, ipilimumab (a CTLA-4 inhibitor), the first immune checkpoint inhibitor, was approved by the FDA (55). From 2011 to 2024, immune checkpoint inhibitors have received continuous approval for marketing and expanded indications (53), such as pembrolizumab (a PD-1 inhibitor) (51), atezolizumab (a PD-L1 inhibitor) (52), and relatlimab (a lymphocyte-activation gene 3 inhibitor) (56). Over the past decade, while immune checkpoint inhibitors have yielded remarkable results in the clinical treatment of tumors (54), they continue to face challenges such as primary drug resistance, secondary drug resistance (38), immune-related adverse events (57), limitations in biomarkers (58), and restrictions on indications (38). These issues are closely linked to the mechanisms underlying tumor immune escape. A comprehensive investigation into the mechanisms of tumor immune escape will provide a scientific foundation for addressing these challenges and will facilitate the further advancement of immunotherapy.

#### 4.3.2 Tumor immune escape and the immune microenvironment

Immune infiltration, natural killer cells, immunogenic cell death, and extracellular vesicles are intricately interconnected within the tumor immune microenvironment. These components collectively modulate the immune response to tumors through their interactions. Immune infiltration refers to the process by which immune cells, such as T cells, B cells, and natural killer cells, migrate into tumor tissues from the peripheral circulatory system (59). This infiltration can facilitate tumor immune escape by establishing an immunosuppressive microenvironment (46). Additionally, there exists a synergistic relationship between immune infiltration and immunogenic cell death, wherein infiltrating natural killer cells can directly eliminate tumor cells and induce the death of immunogenic cells. Immunogenic cell death has the potential to reverse the tumor’s immunosuppressive microenvironment and enhance immune infiltration by activating the immune response (60). Furthermore, immune infiltration and extracellular vesicles are interrelated, as immune infiltration affects the immune response by modifying the formation and function of extracellular vesicles, while extracellular vesicles play a critical role in immune infiltration by regulating the recruitment, activation, and function of immune cells (61). Ultimately, by modulating the process of immune infiltration, it is possible to inhibit tumor immune escape, enhance the efficacy of immunotherapy, and provide greater clinical benefits to cancer patients. Therefore, immune infiltration, natural killer cells, immunogenic cell death, and extracellular vesicles represent emerging frontiers in the study of tumor immune escape.

#### 4.3.3 Tumor immune escape and metabolic reprogramming

In recent years, research on metabolic reprogramming in tumor immune escape has garnered increasing attention. Targeting these metabolic pathways can reverse the immunosuppressive microenvironment, mitigate tumor immune escape, and enhance the efficacy of immunotherapy (62). Ferroptosis, a form of programmed cell death characterized by lipid peroxidation and dysregulated iron metabolism (63), can enhance the anti-tumor immune response and mitigate tumor immune escape by inducing ferroptosis in cancer cells (64). Thus, metabolism and ferroptosis represent emerging frontiers in the study of tumor immune escape. Ferroptosis reveals a novel mechanism of tumor immune escape, which is beneficial for exploring combined treatment strategies that target both metabolism and immunity.

#### 4.3.4 Melanoma and lung adenocarcinoma

Melanoma and lung adenocarcinoma are characterized by a high mutation burden and demonstrate sensitivity to immunotherapy (65, 66). The approval of ipilimumab as the first immune checkpoint inhibitor for the treatment of advanced melanoma in 2011 (55) and the approval of pembrolizumab for the first-line treatment of advanced non-small cell lung cancer in 2015 (67) highlight the significance of immunotherapy in these cancers. Despite the significant efficacy achieved through immunotherapy for these cancers (68), some patients still face the challenge of secondary immune escape during treatment (69). Recent research efforts have increasingly focused on understanding the mechanisms of tumor immune escape (70).

#### 4.3.5 Tumor immune escape and prognosis

Over the past decade, immunotherapy has demonstrated remarkable success in cancer treatment (71). There is a growing interest in the impact of immunotherapy on patient prognosis (72). By delving deeper into the mechanisms of tumor immune escape, the efficacy of immunotherapy can be optimized, leading to improved patient outcomes (73). Prognosis has now emerged as a pivotal frontier in the study of tumor immune escape.

### 4.4 Future directions

The field of tumor immune escape has garnered significant interest from numerous scholars. In light of the identified hotspots and emerging frontiers, future studies on tumor immune escape should focus on elucidating its fundamental mechanisms, developing innovative immunotherapeutic strategies, optimizing patient selection and treatment protocols, investigating the tumor immune microenvironment, identifying novel therapeutic targets and biomarkers, reversing drug resistance, enhancing prognostic evaluations, balancing therapeutic efficacy with potential adverse effects, and advancing precision medicine through improved molecular detection and tailored treatment approaches.

### 4.5 Limitations

This study is subject to several limitations. Firstly, the data source for this study was limited to the WoSCC database. Although the WoSCC database is the most commonly used source for bibliometric analysis, other data sources, such as PubMed and Scopus, also exist. Secondly, the literature selected for analysis was restricted to publications in the English language, which may have introduced a bias related to language. Furthermore, it is noteworthy that earlier publications tend to accumulate a greater number of citations over time. This temporal discrepancy may influence the outcomes of citation and co-citation analyses. Additionally, our manuscript did not address the disambiguation problem with common Chinese names. Lastly, given that the WoSCC database is subject to continuous updates, our study may have inadvertently overlooked some of the most recent research findings.

## 5 Conclusion

This study provides a systematic analysis of the bibliometric characteristics associated with global publications on tumor immune escape. It delineates the current status, research frontiers, and developmental trends in this field.

1. Research on tumor immune escape has garnered significant global interest, with the USA, China, and Germany identified as the most active contributors in this field. Notable international collaboration has been observed between the USA and China.
2. Frontiers in Immunology, Nature Communications, the Journal for ImmunoTherapy of Cancer, and Nature are recognized as the most influential journals for literature related to tumor immune escape.
3. Current hotspot frontiers in tumor immune escape research include immunotherapy, the tumor microenvironment, PD-L1, and PD-1.
4. Emerging frontiers in this field encompass immune checkpoint inhibitors, immune infiltration, natural killer cells, extracellular vesicles, ferroptosis, immunogenic cell death, metabolism, lung adenocarcinoma, melanoma, and prognosis.
5. Future investigations into tumor immune escape should prioritize the optimization of treatment, the identification of biomarkers, the characterization of the tumor microenvironment, the mechanisms of drug resistance, prognostic factors, the balance between efficacy and safety, and the advancement of precision medicine.

## Data Availability

The original contributions presented in the study are included in the article/**Supplementary Material**. Further inquiries can be directed to the corresponding author.
