# Supplementary material for: Bibliometric and visualized analysis of global distribution and research frontiers in tumor immune escape
Source: Front Immunol. 2025 Jun 5;16:1586120. doi: 10.3389/fimmu.2025.1586120 (PMC12176767; doi:10.3389/fimmu.2025.1586120)
Supplement: Supplementary file 1 [file DataSheet1.docx]

Supplement File 1. The complete Boolean search formula used in the WoSCC query.

(((((((((((((((TS=(Neoplasms)) OR TS=(Tumors)) OR TS=(Neoplasia)) OR TS=(Neoplasias)) OR TS=(Neoplasm)) OR TS=(Tumor)) OR TS=(Cancer)) OR TS=(Cancers)) OR TS=(Malignant Neoplasm)) OR TS=(Malignancy)) OR TS=(Malignancies)) OR TS=(Malignant Neoplasms)) OR TS=(Neoplasm, Malignant)) OR TS=(Neoplasms, Malignant)) AND (((((((((((((TS=(Tumor Escape)) OR TS=(Immune Escape, Tumor)) OR TS=(Tumor Immune Escape)) OR TS=(Tumor Immune Evasion)) OR TS=(Evasions, Tumor Immune)) OR TS=(Evasion, Tumor Immune)) OR TS=(Immune Evasions, Tumor)) OR TS=(Tumor Immune Evasions)) OR TS=(Immune Evasion, Tumor)) OR TS=(Immune Evasion)) OR TS=(Evasion, Immune)) OR TS=(Evasions, Immune)) OR TS=(Immune Evasions))) AND DT=(Article)
