# Supplementary material for: Bibliometric and visualized analysis of global distribution and research frontiers in tumor immune escape
Source: Front Immunol. 2025 Jun 5;16:1586120. doi: 10.3389/fimmu.2025.1586120 (PMC12176767; doi:10.3389/fimmu.2025.1586120)
Supplement: Supplementary file 2 [file Table1.docx]

Supplement Table 1. Parameter settings for different bibliometric analyses in the VOSviewer software.

| **Type of analysis** | **Unit of analysis** | **Key Parameters** | **Output** |
| --- | --- | --- | --- |
| Co-authorship | Authors | minimum number of documents of an author: 1;  minimum number of citations of an author: 0. | 71,684 authors |
|  | Institutions | minimum number of documents of an institution: 1;  minimum number of citations of an institution: 0. | 9,254 institutions |
|  | Countries | minimum number of documents of a country: 1;  minimum number of citations of a country: 0. | 121 countries |
| Co-occurrence | Author Keywords | minimum number of occurrences of an author keyword: 1. | 16,465 author keywords |
| Citation | Documents | minimum number of citations of a document: 0. | 11,128 documents |
|  | Journals | minimum number of documents for a journal: 1;  minimum number of citations for a journal: 0. | 1,612 journals |
|  | Authors | minimum number of documents for an author: 1;  minimum number of citations for an author: 0. | 71,684 authors |
|  | Institutions | minimum number of documents for an institution: 1;  minimum number of citations for an institution: 0. | 9,254 institutions |
|  | Countries | minimum number of documents for a country: 1;  minimum number of citations for a country: 0. | 121 countries |
| Bibliographic coupling | Documents | minimum number of citations of a document: 0. | 11,128 documents |
|  | Journals | minimum number of documents for a journal: 1;  minimum number of citations for a journal: 0. | 1,612 journals |
|  | Authors | minimum number of documents for an author: 1;  minimum number of citations for an author: 0. | 71,684 authors |
|  | Institutions | minimum number of documents for an institution: 1;  minimum number of citations for an institution: 0. | 9,254 institutions |
|  | Countries | minimum number of documents for a country: 1;  minimum number of citations for a country: 0. | 121 countries |
| Co-citation | References | minimum number of citations of a cited reference: 1. | 331,872 references |
|  | Cited Journals | minimum number of citations of a cited source: 1. | 14,355 cited journals |
|  | Cited Authors | minimum number of citations of a cited author: 1. | 174,657 cited authors |
