# Supplementary material for: Bibliometric and visualized analysis of global distribution and research frontiers in tumor immune escape
Source: Front Immunol. 2025 Jun 5;16:1586120. doi: 10.3389/fimmu.2025.1586120 (PMC12176767; doi:10.3389/fimmu.2025.1586120)
Supplement: Supplementary file 3 [file Table2.docx]

Supplementary Table 2. The complete details for the top ten most globally or locally cited articles in tumor immune escape research.

| The title of the articles | First author | Year | Journal | IF(2023) | Q |
| --- | --- | --- | --- | --- | --- |
| Pan-cancer Immunogenomic Analyses Reveal Genotype-Immunophenotype Relationships and Predictors of Response to Checkpoint Blockade | charoentong | 2017 | cell rep | 7.5 | Q1 |
| Signatures of T cell dysfunction and exclusion predict cancer immunotherapy response | jiang | 2018 | nat med | 58.7 | Q1 |
| PD-1 Blockade with Nivolumab in Relapsed or Refractory Hodgkin's Lymphoma | ansell | 2015 | new engl j med | 96.3 | Q1 |
| Mutations Associated with Acquired Resistance to PD-1 Blockade in Melanoma | zaretsky | 2016 | new engl j med | 96.3 | Q1 |
| Melanoma-intrinsic β-catenin signalling prevents anti-tumour immunity | spranger | 2015 | nature | 50.5 | Q1 |
| Interferon Receptor Signaling Pathways Regulating PD-L1 and PD-L2 Expression | garcia-diaz | 2017 | cell rep | 7.5 | Q1 |
| Exosomal PD-L1 contributes to immunosuppression and is associated with anti-PD-1 response | chen | 2018 | nature | 50.5 | Q1 |
| Radiation and dual checkpoint blockade activate non-redundant immune mechanisms in cancer | twyman-saint victor | 2015 | nature | 50.5 | Q1 |
| Immune Escape Mechanisms as a Guide for Cancer Immunotherapy | beatty | 2015 | clin cancer res | 10.4 | Q1 |
| PD-1 expression by tumour-associated macrophages inhibits phagocytosis and tumour immunity | gordon | 2017 | nature | 50.5 | Q1 |
| TGFβ drives immune evasion in genetically reconstituted colon cancer metastasis | tauriello | 2018 | nature | 50.5 | Q1 |
| Upregulation of PD-L1 by EGFR Activation Mediates the Immune Escape in EGFR-Driven NSCLC: Implication for Optional Immune Targeted Therapy for NSCLC Patients with EGFR Mutation | chen | 2015 | j thorac oncol | 21.1 | Q1 |
| Enhancing cancer immunotherapy using antiangiogenics: opportunities and challenges | fukurnura | 2018 | nat rev clin oncol | 81.1 | Q1 |
| PD-L1 on tumor cells is sufficient for immune evasion in immunogenic tumors and inhibits CD8 T cell cytotoxicity | juneja | 2017 | j exp med | 12.8 | Q1 |
| cell rep, Cell Reports; nat med, Nature Medicine; new engl j med, New England Journal of Medicine; clin cancer res, Clinical Cancer Research; j thorac oncol, Journal of Thoracic Oncology; nat rev clin oncol, Nature Reviews Clinical Oncology; j exp med, Journal of Experimental Medicine; IF, Impact Factor; Q, Journal Citation Reports Quartile; PD-1, Programmed Death Receptor 1; PD-L1, Programmed Cell Death Ligand 1; PD-L2, Programmed Cell Death Ligand 2; TGFβ, Transforming Growth Factor-β; EGFR, Epidermal Growth Factor Receptor; NSCLC, Non-small Cell Lung Cancer; CD8, Cluster of Differentiation 8. | | | | | |
